# Identification of Host Factors Involved in Human Cytomegalovirus Replication, Assembly, and Egress Using a Two-Step Small Interfering RNA Screen

**DOI:** 10.1128/mBio.00716-18

**Published:** 2018-06-26

**Authors:** Dominique McCormick, Yao-Tang Lin, Finn Grey

**Affiliations:** a Division of Infection and Immunity, The Roslin Institute, University of Edinburgh, Easter Bush, Midlothian, United Kingdom; Columbia University Medical College

**Keywords:** assembly and egress, COPA, COPB2, ERC1, human cytomegalovirus, RAB4B, systematic, coatomer, virology

## Abstract

As obligate intracellular parasites, viruses are completely dependent on host factors for replication. Assembly and egress of complex virus particles, such as human cytomegalovirus (HCMV), are likely to require many host factors. Despite this, relatively few have been identified and characterized. This study describes a novel high-throughput, two-step small interfering RNA (siRNA) screen, which independently measures virus replication and virus production. By combining data from replication and virus production, multiple candidate genes were identified in which knockdown resulted in substantial loss of virus production with limited effect on primary replication, suggesting roles in later stages such as virus assembly and egress. Knockdown of the top candidates, ERC1, RAB4B, COPA, and COPB2, caused profound loss of virus production. Despite COPA and COPB2 being reported to function in the same complex, knockdown of these genes produced distinct phenotypes. Furthermore, knockdown of COPA caused increased expression of viral late genes despite substantial inhibition of viral DNA replication. This suggests that efficient viral genome replication is not required for late gene expression. Finally, we show that RAB4B relocates to the viral assembly compartment following infection with HCMV and knockdown of RAB4B reduces the release of intact virion particles, suggesting that it plays a role in virion assembly and egress. This study demonstrates a powerful high-throughput screen for identification of host-virus interactions, identifies multiple host genes associated with HCMV assembly and egress, and uncovers potentially independent functions for coatomer components COPA and COPB2 during infection.

## IMPORTANCE

Human cytomegalovirus infection is a significant cause of disease in immunocompromised populations, individuals with heart disease, and recipients of solid organ and bone marrow transplants. HCMV is also the leading cause of infectious congenital birth defects. The majority of antivirals in clinical use target components of the virus to specifically inhibit replication. However, a major drawback of this approach is the emergence of resistance. An alternative approach is to target host factors that the virus requires for successful infection. In this study, multiple host factors were identified that were found to be essential for the production of newly infectious human cytomegalovirus. Identifying which host genes are necessary for virus replication extends our understanding of how viruses replicate and how cells function and provides potential targets for novel antivirals.

## INTRODUCTION

Successful generation of virions from infected cells is a complex process requiring orchestrated interactions between the virus and the host cell (1, 2). Following binding and entry of human cytomegalovirus (HCMV), the viral capsid is translocated to the nucleus where the genome is delivered. During acute infection, viral DNA is replicated, is packaged into capsids, and traverses the nuclear membranes into the cytoplasm, where the virus gains its tegument and envelope before being released from the cell as newly infectious virions. While the roles of many cytomegalovirus genes have been characterized, the role of host genes is poorly understood, especially in virus assembly and egress, where relatively few host genes have been identified to be critically involved in this process (3, 4).

Within eukaryotic cells, organelles maintain distinct environments through segregation by lipid membranes. This segregation and the distinct characteristics of cellular organelles are retained, despite highly dynamic and fluid exchange of membrane and constituents between organelles during the normal physiology of the cell. This transport is primarily achieved through vesicles that bud from the donor organelles and deliver both membrane and cargo when they fuse with target membranes (5). The most-studied vesicles are generated through coat protein complexes, such as COPI and COPII, which mediate retrograde and anterograde transport between the endoplasmic reticulum (ER) and Golgi network, and clathrin, which mediates vesicle transport from the plasma membrane and trans-Golgi network (TGN) to early endosomes (6–8). Correct coating, transport, and fusion of vesicles depend on a complex network of regulatory small RAB and ARF GTPases, and their associated binding partners and effectors (9). Many viruses utilize these cellular vesicle pathways for viral protein trafficking while also manipulating the regulation of membrane organization to generate specific structures for virus replication and production (10–12).

Infection with human cytomegalovirus causes a major reorganization of cellular membrane organelles to generate the virion assembly compartment (VAC), a phenomenon particular to the betaherpesviruses. This compartment forms late in infection by concentration of multiple cellular membranes as well as prominent components of the HCMV virion (13–16). It is thought that the VAC is the focal point of viral assembly and that its formation is essential for efficient production of infectious virions (3). The reorganization of cellular membranes is driven by the interaction of specific virus proteins and microRNAs (miRNAs) with cellular genes involved in membrane organization and trafficking. Previous studies have identified a number of individual Rab proteins and interacting factors as being important in the process of HCMV assembly and egress. HCMV glycoprotein M directly interacts with FIP4, a Rab11-interacting protein (17), whereas the tegument protein pp150 requires interaction with Rab6 for trafficking to the VAC (18). Components of the endosomal sorting complex required for transport (ESCRT) have also been identified as playing a role in HCMV maturation (19). Targeting of multiple cellular genes, including VAMP3, RAB5C, RAB11A, SNAP23, and CDC42, by HCMV miRNAs is also important for the formation of the VAC and subsequent virus production (20). More recently, it has been demonstrated that posttranslational modification of Golgi membrane reassembly stacking proteins (Grasp) plays an important role in Golgi fragmentation, VAC formation, and virion production (21). Furthermore, an unconventional myosin, MYO18A, was identified as a component of the VAC using a systematic proteomic approach (22). These cellular factors are likely to be a fraction of the total host-virus interactions that are required for efficient human cytomegalovirus assembly and egress.

To identify additional host-virus interactions involved in assembly and egress, we have devised a high-throughput two-step small interfering RNA (siRNA) screen that measures both primary replication and virus production. Using an siRNA library consisting of pools of four siRNAs targeting 156 host genes involved in membrane organization, we have identified multiple genes required for HCMV replication, assembly, and egress as well as host genes that have a negative impact on HCMV replication. We show that the correlation between primary replication and infectious progeny is far lower than might be expected, indicating that measurement of primary replication alone can be misleading, especially when attempting to identify host targets for antiviral therapy.

## RESULTS

**FIG 1:**
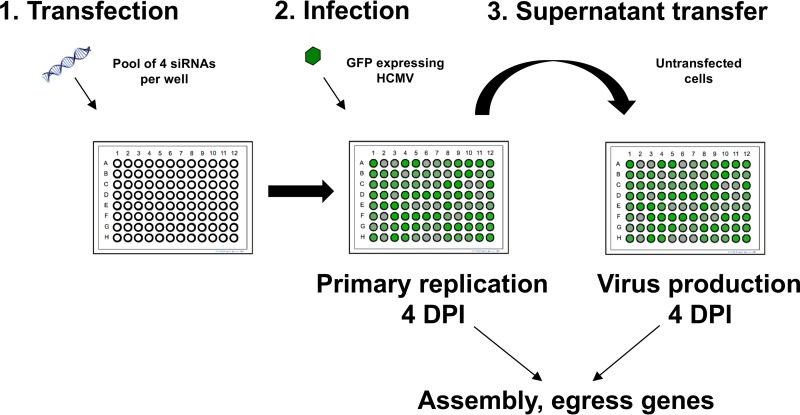
Schematic representation of two-step siRNA screen. Human fibroblast cells were transfected with siRNAs against 156 membrane-associated host genes (1) and infected with the GFP-expressing cytomegalovirus strain TB40/E GFP at 48 h posttransfection (2). Primary replication was tracked by measuring GFP expression. The supernatant, containing progeny virions, was transferred to untransfected cells, and the progress of infection was tracked by measuring GFP expression (3). A comparison of the primary replication at 4 dpi and virion progeny production at 4 dpi enriches for targets predominantly involved in the later stages of the viral life cycle.

Previously, we identified the host gene ATP6V0C as a critical factor in HCMV replication (23). Knockdown of ATP6V0C resulted in modest inhibition of virus replication (as defined by virus gene expression and DNA amplification) but a pronounced reduction in virus progeny, suggesting a defect in assembly or egress. ATP6V0C is a component of the vacuolar ATPase and, among other functions, is involved in membrane organization. To identify additional host membrane organization factors involved in HCMV assembly and egress, we devised a two-step siRNA screen. In brief, using a 96-well format, primary human fibroblast cells were transfected with a pool of 4 siRNAs, each targeting independent sites within a host transcript. The full screen consisted of separate pools targeting 156 host factors. The cells were then infected with a recombinant HCMV expressing green fluorescent protein (GFP), allowing virus entry, translocation to the nucleus, and replication, here referred to as primary replication, to be monitored. GFP levels were monitored for 7 days, and then supernatant was transferred to fresh, untransfected cells where virus progeny was measured by GFP expression. By comparing GFP levels in the first screen at 4 days postinfection (dpi) to the second screen at 4 dpi, host genes potentially involved in assembly and egress can be identified (Fig. 1).

### Correlation between virus replication and virus production based on reporter expression is low.

**FIG 2:**
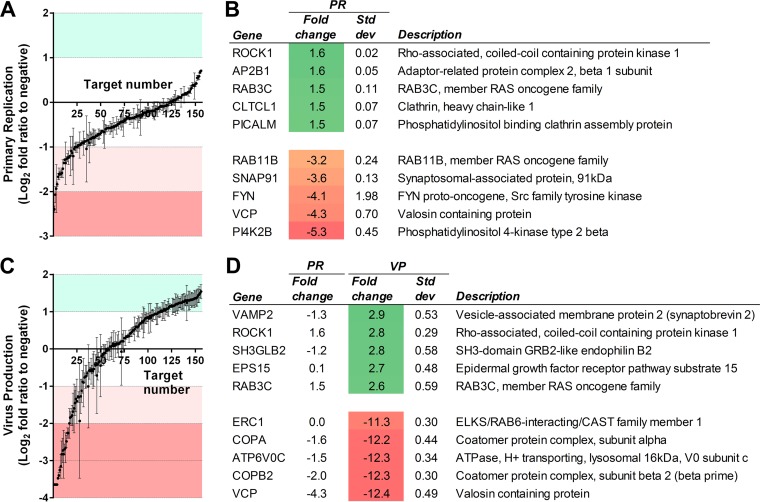
Primary replication (PR) and virus production (VP) are affected by siRNA knockdown of membrane-associated host genes. Relative primary replication of the virus based on GFP fluorescence from the first screen for each of the 156 knockdown experiments is shown in panel A, with the top five proviral and antiviral hits shown in panel B. Relative virus production based on GFP fluorescence from the second screen for each of the 156 knockdown experiments is shown in panel C, with the top five proviral and antiviral hits shown in panel D. All data were normalized to a scrambled siRNA control. *n =* 2; standard deviations shown. PR and VP measured 4 dpi.

**FIG 3:**
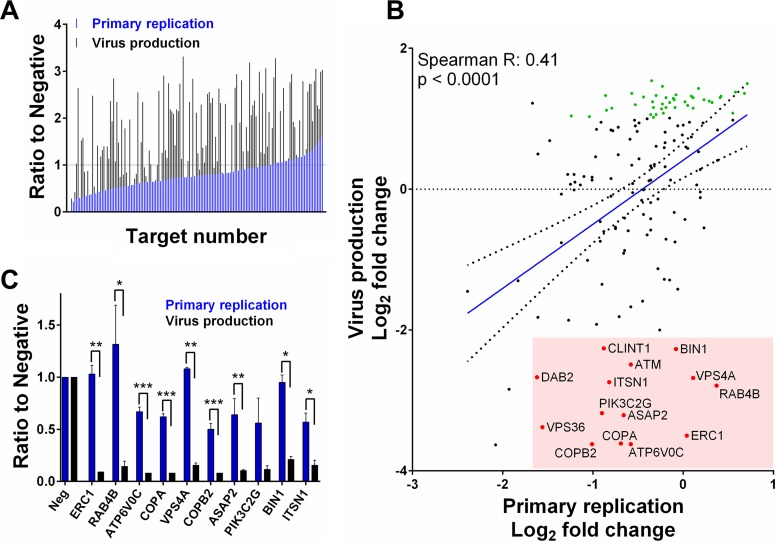
Primary replication is a poor predictor of virus production. Normalized GFP levels from primary replication (first screen, blue) and virus production (second screen, black) ranked by increasing primary replication show that a loss of primary replication does not necessarily lead to a loss of virus production (A). A direct comparison of normalized PR and VP levels was performed to determine correlation and therefore the predictive power of primary replication for virus production. Correlation was relatively low, with a Spearman score of 0.41 (B). This was not due to intrinsic variability of the screen, as correlation between repeats of PR, VP, and the ratio of PR to VP was high (see Fig. S1A, B, and C). Putative assembly and egress hits that show substantially reduced virus production with less than 2-fold effect on virus replication are highlighted in red and labeled, while antiviral hits that would not be identified following a conventional one-step screen with a 2-fold cutoff are shown in green (B). The top 10 putative assembly and egress hits are shown in panel C. *n =* 2; error bars reflect standard deviations. Two-tailed homoscedastic Student’s *t* test was applied to assess whether PR and VP results differed significantly. *P* > 0.05, NS; *P* ≤ 0.05, *; *P* ≤ 0.01,**; *P* ≤ 0.001, ***.

Analysis of the first screen indicated both increased and decreased primary replication ranging from a 5.3-fold reduction in GFP levels compared to negative-control cells following knockdown of PI4K2B to a 1.6-fold increase following knockdown of ROCK1 (Fig. 2A and B; see also Table S1A in the supplemental material). The second screen showed more pronounced effects with virus production ranging from 12.4-fold reduction following valosin-containing protein (VCP) knockdown to a 2.9-fold increase following VAMP2 knockdown, demonstrating divergence between the two screens (Fig. 2C and D; Table S1B). Figure 3A shows GFP levels from the first screen, ranked from lowest to highest, with the corresponding GFP levels for the second screen, showing virus production. Surprisingly, the correlation between primary replication and virus production is lower than would be anticipated given the logical expectation that changes in primary replication will result in corresponding changes in virus production. Pearson correlation analysis, comparing GFP values from the initial screen measuring primary replication with those from the second screen measuring virus production, resulted in an *R* value of only 0.41 (Fig. 3B). The low correlation is not due to intrinsic variability in the system, as correlation between biological repeats of the assay was high (Fig. S1A to C). Of the 29 hits identified in the first screen that showed greater than 2-fold reduction in primary replication, only 11 showed a corresponding decrease in virus production (Fig. S2). Of the 31 hits identified in the second screen that showed greater than 2-fold decrease in virus production, 20 were not identified in the first screen. Given that virus production is the important factor for the identification of potential drug targets, these results suggest that a conventional one-step screen would generate multiple false-positive and -negative results. Furthermore, while the first screen did not identify any host genes resulting in greater than 2-fold increase in primary replication, the second screen identified 48 hits showing greater than 2-fold increase in virus production, demonstrating greater sensitivity of the second screen for identification of antiviral host genes (shown in green in Fig. 3B; see also Table S2).

10.1128/mBio.00716-18.1 FIG S1  Correlation analysis between replicates of the screens shows a high level of reproducibility. Normalized GFP levels of biological repeats correlated well when comparing primary replication (A) (Spearman *R*, 0.87), virus production (B) (Spearman *R*, 0.94), and the ratio between virus production and primary replication (C) (Spearman *R*, 0.94). Download FIG S1, TIF file, 0.8 MB. Copyright © 2018 McCormick et al. 2018 McCormick et al. This content is distributed under the terms of the Creative Commons Attribution 4.0 International license.

10.1128/mBio.00716-18.2 FIG S2  Primary replication does not reliably predict virus production. Venn diagram showing the overlap between first screen, measuring primary replication, and second screen, measuring virus production. A total of 29 hits showed greater than 2-fold reduction in primary replication. Thirty-one hits showed greater than 2-fold reduction in virus production. Only 11 hits corresponded between the screens, showing a potentially high level of false-positive and -negative results using a conventional one-step screen that measures only virus replication. Download FIG S2, TIF file, 1.1 MB. Copyright © 2018 McCormick et al. 2018 McCormick et al. This content is distributed under the terms of the Creative Commons Attribution 4.0 International license.

10.1128/mBio.00716-18.8 TABLE S1  (A) Primary replication following siRNA treatment against 156 membrane-associated genes. Relative primary replication of TB40/E GFP in human fibroblast cells at 4 dpi is shown for each of 156 membrane-associated genes inhibited by siRNA treatment. GFP levels were normalized to a negative-control siRNA, and genes were ranked by decreasing effect on primary replication. *n =* 2; Std dev, standard deviations. (B) siRNA knockdown of membrane-associated genes resulted in the loss of virus production. The effect of siRNA treatment on primary replication and virus production relative to a negative control is shown, ranked by decreasing negative effect on virus production. *n =* 2; Std dev, standard deviations. Download TABLE S1, PDF file, 0.1 MB. Copyright © 2018 McCormick et al. 2018 McCormick et al. This content is distributed under the terms of the Creative Commons Attribution 4.0 International license.

10.1128/mBio.00716-18.9 TABLE S2  An increase in virus production ensued from siRNA knockdown of some membrane-associated genes. The fold change in primary replication and virus production relative to the negative control is shown, ranked by decreasing positive effect on virus production. *n =* 2; Std dev, standard deviations. Download TABLE S2, PDF file, 0.2 MB. Copyright © 2018 McCormick et al. 2018 McCormick et al. This content is distributed under the terms of the Creative Commons Attribution 4.0 International license.

### Identification of candidate host factors involved in assembly and egress.

Knockdown of multiple host genes resulted in more pronounced inhibition of virus production than of primary replication based on reporter expression levels, suggesting a potential role in later stages of virus replication, such as assembly and egress (Fig. 3B; labeled hits highlighted in red). The top 10 assembly and egress candidates based on the ratio between primary replication and virus production are shown in Fig. 3C. ATP6V0C and VSP4A, previously identified as host factors involved in assembly and egress (4, 23), are present within the top 10 hits showing substantially greater effects on virus production than on primary replication. VPS36, a component of the ESCORTII complex which has previously been linked to HCMV assembly and egress, is also in the top 20 hits (Table S3). These data provide confidence that this is an effective strategy for identifying host factors involved in assembly and egress. Knockdown of several host factors demonstrated a similar profile, with a greater decrease in virus production than in primary replication, suggesting the identification of novel host factors potentially involved in assembly and egress (Fig. 3C; Table S3). The top four novel candidates, ERC1, RAB4B, COPA, and COPB2, were taken forward for further validation and characterization.

10.1128/mBio.00716-18.10 TABLE S3  The ratio between virus production and primary replication following siRNA treatment against 156 membrane-associated genes. Genes were ranked by increasing VP/PR ratio to identify hits that had a larger effect on virus production than on primary replication. Two-tailed homoscedastic Student’s *t* test was applied to assess whether primary replication and virus production results differed significantly. *P* > 0.05, NS; *P* ≤ 0.05, *; *P* ≤ 0.01, **; *P* ≤ 0.001, ***; *P* ≤ 0.0001, ****. Download TABLE S3, PDF file, 0.1 MB. Copyright © 2018 McCormick et al. 2018 McCormick et al. This content is distributed under the terms of the Creative Commons Attribution 4.0 International license.

### Knockdown of candidate genes results in profound loss of virus production.

It is essential to validate each of the siRNA assays to confirm that the observed phenotype is due to specific knockdown and not due to “off-target” effects. Each siRNA pool contains four independent siRNAs that target different sequences within the target transcript. Deconvoluting the siRNA pools allows validation of target knockdown with each of the four siRNAs individually. All four individual siRNAs against COPA and COPB2 generated the same or similar phenotypes as the siRNA pools (Fig. S3A). Three of four siRNAs phenocopied the results for RAB4B, while two of four siRNAs against ERC1 showed similar phenotypes as the pooled siRNAs (Fig. S3A). While knockdown of gene expression, as measured by reverse transcription-quantitative PCR (qRT-PCR), correlated with the phenotype for COPA, COPB2, and RAB4B, all four deconvoluted siRNAs caused knockdown of ERC1 with only two resulting in the same phenotype as the pooled siRNA. However, the ERC1 genomic region is highly complex, with 25 alternative transcripts being generated with only three coding for protein, complicating the interpretation of the data. Therefore, ERC1 protein levels were measured by Western blot analysis following transfection with the individual deconvoluted siRNAs. Analysis of protein levels correlated with the observed phenotype, indicating that the individual siRNAs validate the result from the pooled siRNAs (Fig. S3B and C).

10.1128/mBio.00716-18.3 FIG S3  Confirmation of phenotypes using deconvoluted siRNAs. (A) Primary fibroblast cells were transfected with the 4 individual deconvoluted siRNAs for the top assembly and egress hits to rule out potential off-target effects. Total RNA was extracted 48 h posttransfection, and transcript levels were measured by qRT-PCR analysis to determine knockdown efficiency. In parallel, cells were infected with TB40/E GFP 48 h posttransfection, and GFP levels were measured using the two-step screen to determine primary replication and virus production. Knockdown efficiency was compared to effects on virus replication and virus production. (B) Western blot analysis showing ERC1 protein levels following knockdown with the pooled siRNA (P) or deconvoluted siRNAs A, B, C, and D. These were compared to cells transfected with a negative-control siRNA. (C) Quantification of protein levels compared to effects on primary replication and virus production. siRNAs B and D result in greater knockdown of ERC1 and a correspondingly higher reduction in virus production. (D) Fibroblast cells were transfected with pooled siRNAs against indicated cellular targets and cell viability was measured 48 h posttransfection using CellTiter-Blue. *n =* 2; error bars represent standard deviations. Download FIG S3, JPG file, 0.5 MB. Copyright © 2018 McCormick et al. 2018 McCormick et al. This content is distributed under the terms of the Creative Commons Attribution 4.0 International license.

**FIG 4:**
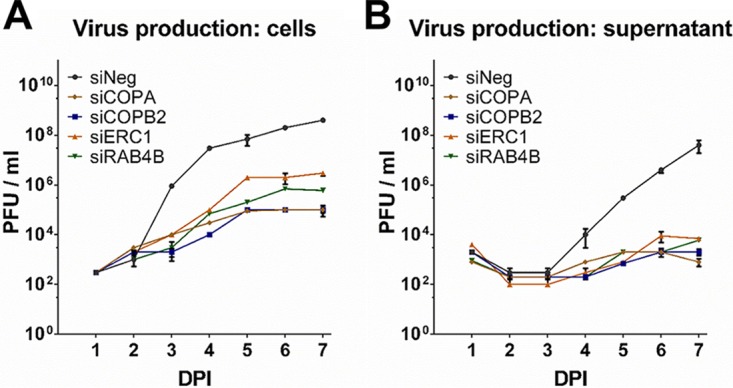
Knockdown of putative assembly and egress hits result in substantial loss of virus replication. Fibroblast cells were transfected with pooled siRNAs against the indicated targets and infected 48 h posttransfection with TB40/E GFP at an MOI of 3. Supernatant and cells were harvested at the indicated times postinfection, and virus levels were determined by plaque assay (A and B). *n =* 2; error bars represent standard deviations.

To confirm that viral reporter expression levels correspond to virus replication and production, single-step growth curves were performed following knockdown of the host factors. Cell-associated and supernatant virus levels were determined following high-multiplicity infection (multiplicity of infection [MOI] of 3) by plaque assay (Fig. 4A and B). In all four cases, knockdown of the target gene resulted in significant decrease in virus production compared to cells transfected with a negative-control siRNA. This was particularly evident in supernatant virus where levels were reduced by between 4 and 5 logs and there was little evidence of substantial virus production throughout the time course. Knockdown of the four candidate genes did not result in toxicity as determined by cell viability assay (Fig. S3D).

### Loss of virus production is not due to major defects in virus entry.

**FIG 5:**
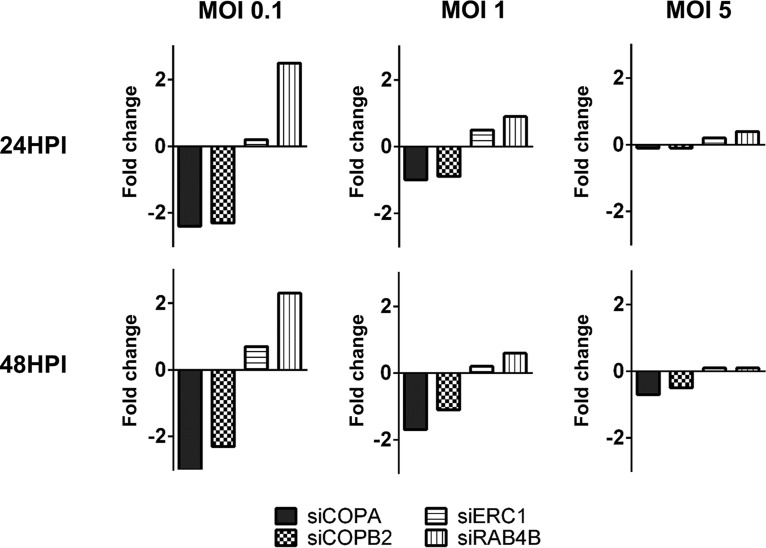
Inhibition of COPA, COPB2, ERC1, or RAB4B does not prevent virions from trafficking to the nucleus. Primary human fibroblast cells were transfected with indicated siRNAs and infected 48 h posttransfection with TB40/E GFP at MOIs of 0.1, 1, and 5. Cells were harvested 24 and 48 HPI, and GFP levels were measured by flow cytometry analysis to determine the effects on virus entry and genome translocation to the nucleus. Fold change in GFP-positive cells relative to cells transfected with a scrambled negative control is shown.

We next wanted to define the stage at which the defect in virus production was occurring following knockdown of each of the identified host genes. To determine the effect on virus entry and translocation to the nucleus, viral GFP levels were measured by flow cytometry analysis following knockdown of the candidate host genes. Cells were infected at high, medium, and low multiplicity, and GFP levels were measured at 24 h postinfection (hpi) and 48 hpi (Fig. 5 and S4). Consistent with the data from the first screen, knockdown of ERC1 had little effect on GFP levels at early times postinfection, while knockdown of COPA and COPB2 caused approximately 2-fold reduction of GFP signal at low multiplicity of infection, suggesting reduced efficiency in virus entry or translocation. These effects could also be visualized by fluorescence microscopy (Fig. S5). While this may contribute to the observed phenotype, it is unlikely to fully account for the substantial loss in virus production, especially as the effects are overcome at higher multiplicities, such as those used for the growth curve analysis. In contrast to COPA and COPB2, knockdown of RAB4B resulted in a consistent increase in GFP signal at lower multiplicities. While RAB4B is clearly necessary for successful virus production, these results paradoxically suggest that RAB4B activity hinders early events in HCMV infection.

10.1128/mBio.00716-18.4 FIG S4  Inhibition of COPA, COPB2, ERC1 or RAB4B does not prevent virions from trafficking to the nucleus. Primary human fibroblast cells were transfected with indicated siRNAs and infected 48 h posttransfection with TB40/E GFP at MOIs of 0.1, 1 and 5. Cells were harvested 24 and 48 HPI and GFP levels were measured by flow cytometry analysis to determine the effects on virus entry and genome translocation to the nucleus. Fluorescence is shown as MFI. Download FIG S4, JPG file, 0.9 MB. Copyright © 2018 McCormick et al. 2018 McCormick et al. This content is distributed under the terms of the Creative Commons Attribution 4.0 International license.

10.1128/mBio.00716-18.5 FIG S5  siRNA knockdown of COPA, COPB2, ERC1, or RAB4B does not prevent the translocation of TB40/E GFP DNA to the nucleus. Fluorescence microscopy analysis of human fibroblast cells transfected with siRNAs against COPA, COPB2, RAB4B, ERC1, or a negative-control siRNA. Cells were infected at an MOI of 3 with TB40/E GFP and imaged at 1 and 2 dpi. Download FIG S5, JPG file, 2.3 MB. Copyright © 2018 McCormick et al. 2018 McCormick et al. This content is distributed under the terms of the Creative Commons Attribution 4.0 International license.

### Knockdown of ERC1, COPA, and COPB2 results in reduced viral DNA replication.

**FIG 6:**
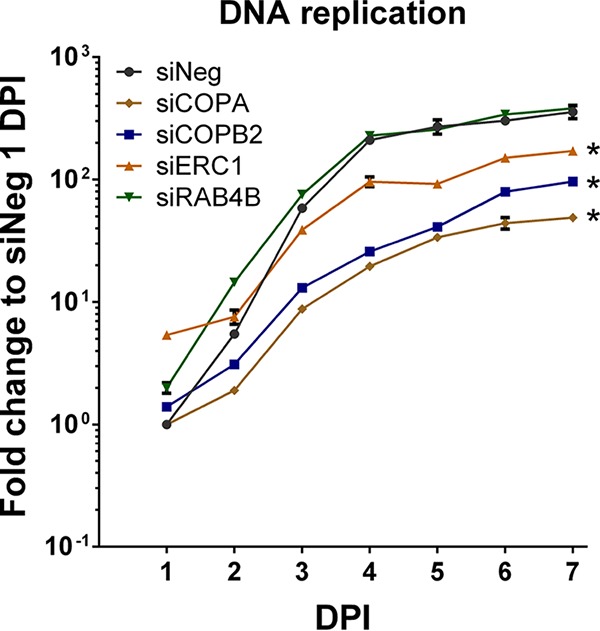
Knockdown of COPA, COPB2, and ERC1, but not RAB4B, results in reduced viral DNA replication. Fibroblast cells were transfected with siRNA pools against indicated cellular targets or a control scrambled siRNA and infected with TB40/E GFP at an MOI of 3 at 48 h posttransfection. Total genomic DNA was isolated at the indicated time points, and viral genome levels were determined by qPCR. Inhibition of COPA and COPB2 resulted in a 7-fold and 4-fold loss of viral DNA replication by 7 DPI, respectively. A 2-fold loss of DNA replication was observed after siERC1 treatment. However, inhibition of RAB4B did not affect viral DNA replication. *n =* 2; error bars show standard deviations. *, *P* value < 0.05 determined by analysis of variance two-way test.

To determine whether viral genome amplification was affected, viral DNA levels were analyzed by quantitative PCR (qPCR) following siRNA knockdown of RAB4B, ERC1, COPA, and COPB2. Knockdown of COPA and COPB2 resulted in approximately 10-fold reductions in viral genome amplification (Fig. 6). Knockdown of ERC1 also resulted in reduced viral DNA replication but not to the same extent as knockdown of the COPI subunits. Knockdown of RAB4B had little effect on viral DNA replication, suggesting that the major defect following RAB4B knockdown occurs after DNA amplification.

### Knockdown of COPA results in specific increase in viral late gene expression.

**FIG 7:**
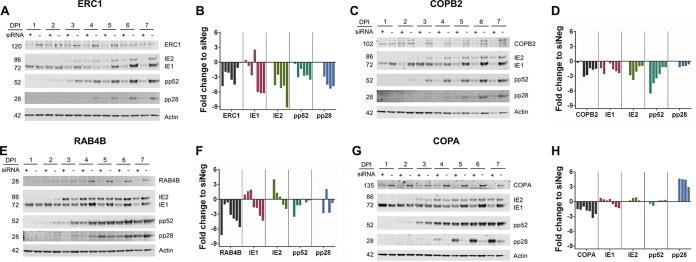
Knockdown of COPA results in increase of pp28 protein expression. Fibroblast cells were transfected with siRNA pools against the indicated cellular targets (+) or a negative-control scrambled siRNA (−) and infected 48 h posttransfection with TB40/E GFP at an MOI of 3. Total protein was harvested, and levels of immediate early (IE1 and IE2), early (pp52), and late (pp28) proteins were monitored over 7 days postinfection (dpi) by Western blot analysis. ERC1 and COPB2 knockdown resulted in decreased levels of all three classes of viral protein (A to D). Knockdown of RAB4B resulted in an initial increase in IE protein expression. Expression of early and late proteins was not dramatically reduced (E and F). COPA inhibition did not affect IE or E proteins but led to a large increase in L protein levels (G and H).

**FIG 8:**
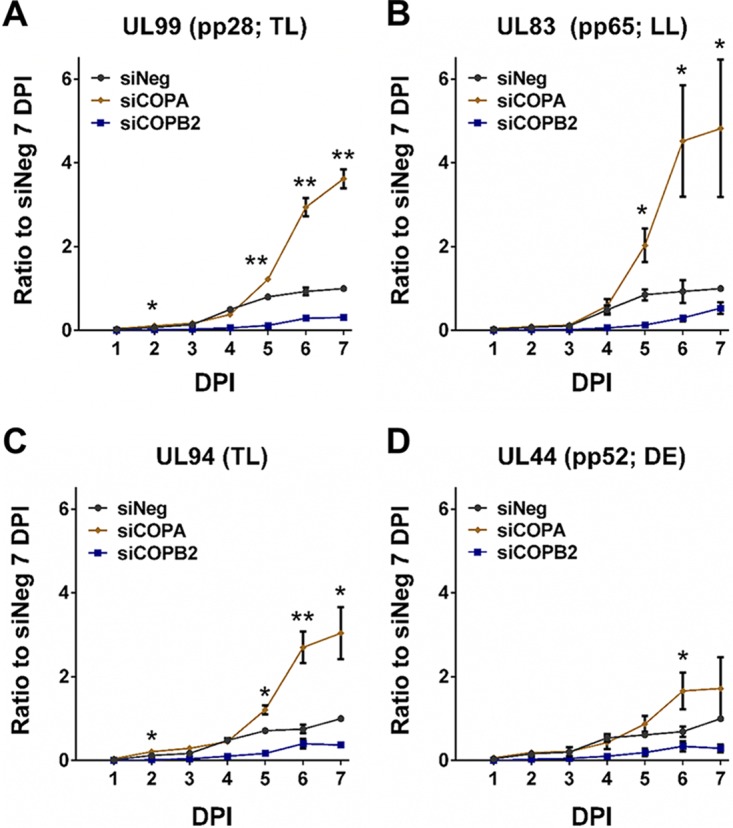
COPA inhibition results in increased late gene transcription. Fibroblast cells were transfected with siRNA pools against COPA, COPB2, or a negative-control scrambled siRNA and infected with TB40/E GFP 48 h posttransfection at an MOI of 3. Total RNA was harvested at the indicated time points, and relative viral transcript levels were determined by qRT-PCR for UL99 (pp28) (A), UL83 (pp65) (B), UL94 (C), and UL44 (pp52). Expression levels were normalized to GAPDH and expressed relative to the negative-control levels at the 7-day time point. *n =* 2; error bars show standard deviations. Two-tailed homoscedastic Student’s *t* test was applied to assess whether siCOPA treatment resulted in significantly different RNA levels compared to negative-control levels. *P* > 0.05, NS; *P* ≤ 0.05, *; *P* ≤ 0.01, **.

To further characterize the role of the identified host genes in HCMV replication, viral protein expression was monitored by Western blot analysis. HCMV gene expression occurs through a tightly regulated cascade mechanism with genes defined by kinetic class, including immediate early, early, and late. Protein levels representing each kinetic class (IE1 and IE2, immediate early; pp52, early; pp28, late) were monitored following knockdown of RAB4B, ERC1, COPA, and COPB2 (Fig. 7). Knockdown of ERC1 and COPB2 resulted in reduced levels of all three classes of viral proteins (Fig. 7A to D). Knockdown of RAB4B resulted in an initial increase in immediate early expression, corresponding with the increase in GFP expression at early time points and supporting the notion that RAB4B has a negative impact on early events in virus infection. Early and late protein expression were not drastically impacted, consistent with a classic assembly and egress phenotype (Fig. 7E and F). Despite substantial reductions in DNA amplification, knockdown of COPA did not cause a defect in immediate early and early protein expression, while levels of the late protein, pp28, increased dramatically (Fig. 7G and H). This result is surprising as both COPA and COPB2 are components of the same COPI complex and therefore knockdown of either would be expected to produce the same phenotype. Furthermore, current models suggest that late gene expression is tightly linked to viral DNA replication. One possible explanation is that knockdown of COPA results in the accumulation of pp28 protein rather than increased transcription. To determine whether the increase in pp28 levels is due to protein accumulation or increased transcription, mRNA levels were determined by qRT-PCR analysis (Fig. 8A). The results clearly show that knockdown of COPA results in a dramatic increase in UL99 (pp28) mRNA levels, suggesting that the increase in pp28 levels is due to increased gene expression. To determine whether this is specific to pp28 or common to late genes, UL83 (pp65, true late), UL94 (late), and UL44 (pp52, delayed early) RNA levels were determined by qRT-PCR. Knockdown of COPA results in a clear and dramatic increase in late gene RNA levels 5 days postinfection (Fig. 8A to C). This appears to be specific to late genes, as RNA levels of the early gene UL44 (pp52) did not dramatically increase (Fig. 8D). To confirm that the effect is specific to COPA knockdown, RNA levels were tested following knockdown with each of the deconvoluted siRNAs, which all showed the same increase in UL99 (pp28) RNA levels, confirming that this effect is specific to COPA knockdown (Fig. S6).

10.1128/mBio.00716-18.6 FIG S6  Correlation of reduced COPA mRNA levels with increased pp28 mRNA levels. Primary fibroblast cells were transfected with 4 individual COPA deconvoluted siRNAs, a pool of COPA siRNAs, or a negative-control siRNA and infected 48 h posttransfection with TB40/E GFP at an MOI of 3. Total RNA was extracted 6 dpi, and transcript levels were measured by qRT-PCR analysis. The normalized levels of pp28 RNA were found to increase with a corresponding decrease in the normalized levels of COPA RNA (Spearman *R*, 0.68). Download FIG S6, JPG file, 0.3 MB. Copyright © 2018 McCormick et al. 2018 McCormick et al. This content is distributed under the terms of the Creative Commons Attribution 4.0 International license.

### RAB4B relocates to the viral assembly compartment.

RAB4B was selected for more detailed characterization as knockdown resulted in effects most closely resembling a clear assembly and egress phenotype, with little effect on viral DNA replication or protein expression while demonstrating a severe defect in the production of infectious virus. During infection with HCMV, the virus causes a major reorganization of intracellular membranous organelles to form a virus-specific structure known as the VAC. The VAC comprises a perinuclear inclusion, derived from trans-Golgi network vacuoles, early endosomes, and vacuoles bearing markers of the ESCRT III machinery (13–16). The current model for HCMV virion assembly suggests that viral capsids are transported from the nucleus to the VAC, where the virus acquires its tegument and outer membrane containing viral glycoproteins, before egress to the plasma membrane and release into the extracellular space. To determine the cellular localization of RAB4B during HCMV infection, fibroblast cells were infected with HCMV at a high MOI and fixed 120 hpi. Cells were stained for RAB4B, the viral tegument protein pp28 (which localizes to the VAC), with nuclei stained with 4′,6-diamidino-2-phenylindole (DAPI). Control uninfected cells were also stained for comparison. In uninfected cells, punctate RAB4B staining can be observed throughout the cytoplasm, particularly concentrated around the nucleus (Fig. S7). In infected cells, RAB4B staining clearly colocalizes with pp28 in the VAC, demonstrating relocation of RAB4B to the VAC at late stages of HCMV infection.

10.1128/mBio.00716-18.7 FIG S7  RAB4B relocates to the VAC at late stages of HCMV infection. Fibroblast cells were infected with HCMV and fixed 120 hpi. Cells were stained for the viral tegument protein pp28 (red), which colocalizes with the VAC, RAB4B (green), and DAPI (blue). Punctate staining throughout the cytoplasm and particularly around the nucleus can be seen in uninfected cells. By 120 hpi, RAB4B staining relocates to the VAC, colocalizing with the viral tegument protein pp28. Download FIG S7, JPG file, 2.5 MB. Copyright © 2018 McCormick et al. 2018 McCormick et al. This content is distributed under the terms of the Creative Commons Attribution 4.0 International license.

### Knockdown of RAB4B reduces levels of protected viral genomes in the supernatant.

**FIG 9:**
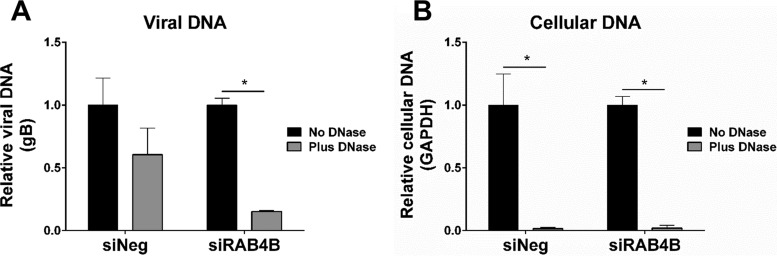
Knockdown of RAB4B reduces levels of protected viral genomes in the supernatant. Fibroblast cells were transfected with RAB4B siRNA or a control scrambled siRNA and then infected 48 h posttransfection with HCMV at a high MOI. Supernatant was harvested 168 hpi, and viral particles were purified by ultracentrifugation. The virus particle was resuspended, and half the sample was treated with DNase before DNA isolation. Viral (A) (primers against gB) and cellular (B) (primers against GAPDH) DNA levels were then determined by qPCR. Data represent two biological repeats with error bars showing standard deviations. Two-tailed homoscedastic Student’s *t* test was applied to assess significance. *P* ≤ 0.05, *.

To determine the effect on virion particle production, supernatant levels of virion-protected viral genome were determined using a DNase protection assay. Fibroblast cells were transfected with an siRNA against RAB4B or a negative-control scrambled siRNA. Forty-eight hours posttransfection, cells were infected at a high MOI with HCMV. Twenty-four hours postinfection, inoculum was removed and cells were washed three times with phosphate-buffered saline (PBS). Supernatant was harvested from the cells 168 hpi, and HCMV virions were isolated by ultracentrifugation. The virion pellet was then resuspended in DNase buffer and split into two samples, one treated with DNase, the other untreated. DNA was then extracted from each sample, and viral DNA levels were measured by qPCR. Viral DNA within intact virion particles would be protected from DNase degradation, whereas viral DNA released from lysed cells would be degraded. As shown in Fig. 9, knockdown of RAB4B resulted in reduced levels of protected viral DNA compared to control cells, suggesting a reduction in the number of intact virions released into the supernatant. However, protected viral genomes were still detected, suggesting that while reduced, DNA-containing virion particles are still released into the supernatant from RAB4B knockdown cells. Levels of cellular DNA, measured using glyceraldehyde-3-phosphate dehydrogenase (GAPDH) primers, were reduced to almost background levels, showing that the DNase treatment was effective in both samples (Fig. 9). These results indicate that knockdown of RAB4B likely affects efficient virion egress or release and does not completely block this process, although released particles may not be infectious.

## DISCUSSION

Viruses are obligate intracellular pathogens that are completely dependent on the host cellular machinery for successful replication. Investigating these interactions will help us understand how the virus replicates, identify potential antiviral targets, and inform our understanding of basic cellular biology. High-throughput systematic screens provide us with a powerful approach for identifying host factors that play important roles during virus infections (24). Here, we developed a two-step siRNA screening method that measures both primary replication and virus production, using a fluorescent reporter virus.

Our results demonstrate that correlation between primary replication and virus production is relatively low, suggesting that conventional single-step screens can result in significant numbers of false positives and false negatives. In some cases, the lack of correlation could be due to the use of a reporter construct driven by an artificial promoter, such as the simian virus 40 (SV40) promoter used in this screen. Factors that have a direct effect on the SV40 promoter may not have relevance to HCMV replication. This can be circumvented by using reporter viruses containing fluorescent proteins directly fused to virion components. However, as shown with the results for COPA knockdown, viral protein levels do not necessarily correlate with infectious virus production. Low-multiplicity infections can also be used; however, such screens do not differentiate between phenotypic effects at different stages. Furthermore, this could be impractical with slower-replicating viruses such as HCMV as siRNA knockdown may not be sustained for the full length of the experiment.

In our relatively small screen of 156 genes, we identified 14 proviral hits and 48 antiviral hits that would not have been identified using a single-step screen with a 2-fold cutoff. For example, knockdown of RAB4B resulted in consistent increased fluorescent reporter expression in the first screen, suggesting an antiviral effect. However, virus production was almost completely abolished. Again, knockdown of VAMP2 resulted in a reduction in fluorescent reporter expression in the first screen but resulted in greater than 2-fold increase in virus production. These results demonstrate the importance of measuring both primary replication and virus production and the advantage of the two-step screen over conventional single-step screens.

The two-step strategy also allowed us to specifically identify genes potentially involved in assembly and egress. By comparing the data sets, we were able to identify host genes which caused little or no reduction in primary replication but significantly reduced virus production. The top four novel genes identified by our screen were ERC1, RAB4B, COPA, and COPB2.

Knockdown of ERC1 caused no effect on viral entry or translocation of the viral genome based on reporter gene expression but did result in reduced genome amplification and viral gene expression in all three temporal classes. ERC1, also known as ELKS due to enriched levels of glutamate, leucine, lysine, and serine, is a multifunctional coiled-coil domain protein linked to both vesicle transport and NF-κB activation (25–29). Through its interaction with RAB6, ERC1 is linked to vesicle transport from the Golgi complex to the cell surface and has been studied extensively in neurotransmitter release (26, 27). ERC1 has previously been linked to production of herpes simplex virus 1 through a RAB6-dependent mechanism, and studies have linked RAB6 with assembly of HCMV through its trafficking of pp150 to the virion assembly compartment, suggesting a possible mechanism for ERC1 in HCMV (18, 30). We also observed reduced virus production following RAB6 knockdown but not to the same extent as the reduction following ERC1 knockdown. This would suggest additional functional roles for ERC1 in HCMV replication and virus production. In addition to roles in membrane organization, ERC1 plays a role in the DNA damage response (25, 28, 29). Genotoxic stress leads to activation of ATM, which, in complex with NEMO, triggers ubiquitination of ERC1. This in turn results in activation of IκB kinase (IKK), degradation of IKBA, and activation of NF-kB. Previous studies have shown that ATM signaling is required for efficient HCMV replication, and knockdown of ATM resulted in a similar phenotype as knockdown of ERC1 in our screen, supporting a role for ERC1 in proviral DNA damage signaling during HCMV infection (31). Studies are ongoing to define whether either or both ERC1 pathways are involved in HCMV replication.

Like all four host genes characterized, knockdown of RAB4B results in a profound defect in production of infectious virus. Surprisingly, knockdown also results in a reproducible increase in early GFP reporter expression in the first screen, suggesting increased levels of infection or translocation and a possible antagonistic role for RAB4B activity in the initial stages of virus infection. This was particularly apparent at low multiplicities of infection. Consistent with this, immediate early protein levels were also higher at early times after infection. Early and late protein expression levels were relatively unaffected, and viral genome amplification was unaffected, suggesting a relatively late defect in virus production. RAB proteins are highly conserved small GTPases that both direct membrane organization and act as specific landmarks, thereby defining membrane organelle localization (9). They function by cycling between GDP-inactive and GTP-active states. When activated, they are anchored to membranes and can bind interacting proteins that modulate their function. RAB4 localizes to specific regions of the early endosome, largely colocalizing with early endosomal antigen 1 (EEA1) (32). The early endosome represents a major crossroads where endocytosed cargo is recycled to the plasma membrane, transported to the TGN, or redirected to lysosomes for degradation (33). Previous studies have reported that RAB4 is essential for recruitment of adapter complexes AP1, AP3, and GGA3, which mediate transport between the early endosome and TGN (32). Markers of early endosomes, such as EEA1 and the TGN, colocalize at late stages of HCMV replication to form the VAC (14, 16). Here, we show that RAB4B also colocalizes to the VAC, suggesting that RAB4B may play a role in virus assembly or the correct formation of the VAC. Furthermore, DNase protection assays indicate that knockdown of RAB4B also reduced the number of genome-containing virions in the supernatant, implying a role in virion egress and release.

In contrast to RAB4B, knockdown of COPA and COPB2 resulted in a 2-fold reduction in GFP expression in the first screen, suggesting decreased entry, translocation, or reporter gene expression. This defect could be overcome using a high MOI. Although early stages of infection may be affected by COPA and COPB2 knockdown, this would not account for the substantial loss in infectious virus production. Instead, knockdown of both resulted in substantial loss of virus genome amplification, suggesting an earlier defect compared to knockdown of RAB4B and ERC1. COPA and COPB2 are subunits of the coatomer complex which forms the outer protein shell of vesicles mediating transport between the ER and Golgi complex (7). There are two main coatomer complexes, COPI and COPII (6, 8). COPII mediates anterograde transport from the ER to the Golgi complex, whereas COPI mediates retrograde transport from the Golgi complex to the ER and between Golgi cisternae. Our results suggest that COPI transport is required for efficient viral genome replication. HCMV DNA replication requires a core of viral proteins, including UL54 (DNA polymerase), UL44 (polymerase accessory protein), UL57 (single-stranded DNA-binding protein), UL105 (helicase), UL70 (primase), and UL102 (primase-associated factor) (34). Additional viral proteins have been shown to augment replication of the HCMV genome (35). These proteins must localize to the nucleus, and knockdown of COPI proteins may disrupt the transport of one or more key viral proteins to the nucleus. COPI subunits have recently been shown to interact with pUL103, a viral protein linked with VAC formation and production of infectious virus (36). Failure in transport of virus proteins to the nucleus may also explain the reduction in viral gene expression, as key viral transactivators must localize to the nucleus.

Despite being part of the same complex, our results suggest that COPA and COPB2 may have independent functions, as knockdown of the individual subunits resulted in clearly distinct phenotypes. While both resulted in reduced viral genome amplification, only knockdown of COPA resulted in a specific increase in viral late gene expression.

Late gene expression is intrinsically linked to viral DNA replication. Recent studies suggest that late gene expression is dependent on an unconventional TTAT transcriptional initiation complex and activation of the lytic origin of replication (OriLyt) (37). Further, it is suggested that late gene expression occurs only from newly synthesized genomes rather than parental genomes from infecting virions. Here, we show that knockdown of COPA substantially reduces viral DNA replication but results in a specific increase in viral late gene expression with little effect on immediate early or early gene expression. This result suggests that while OriLyt activation and initiation of DNA replication may be required for late gene expression, efficient amplification of viral DNA is not required for viral late gene expression. Why knockdown of COPA results in increased late gene expression is not clear. Either COPA is directly involved in negative regulation of late gene expression, or more likely, knockdown of COPA results in an indirect effect on the progression of virus replication which leads to dysregulation of late gene expression.

While further analysis will be required to fully characterize how these host factors are involved in HCMV replication and virus production, the results demonstrate the effectiveness and potential of using a two-step screen over more conventional single-step screens. This study represents a relatively small number of host factors (156), and larger screens using the two-step method are likely to identify additional host factors involved in assembly and egress as well as virus replication and antiviral functions. Furthermore, given that the majority of genome-wide siRNA screens against viruses have employed a conventional single-step approach, using a two-step approach as described here may prove to be more sensitive, reliable, and informative.

## MATERIALS AND METHODS

### HCMV production.

Primary normal human dermal fibroblast (NHDF) cells (Clonetics) were grown in Dulbecco’s modified Eagle’s medium containing 10% fetal calf serum and 1% penicillin–streptomycin–l-glutamine. HCMV strain TB40/E GFP has a GFP cassette driven by an SV40 promoter inserted in the intragenic region between TRS1 and US34 and was obtained from F. Goodrum (38). Strain AD169 was obtained from Jay Nelson. Both strains were grown in primary fibroblast cells after infection at a low MOI. Cells were harvested and cleared, and virus particles were isolated over a 10% sorbitol gradient as previously described (39).

### Human membrane trafficking gene siRNA screen.

The Human siGENOME siRNA Library, which targets 140 membrane trafficking genes (4 siRNAs per gene; Dharmacon, Inc.), and 16 other selected genes were included in the primary screen. In brief, NHDFs were seeded in a 96-well plate a day before siRNA transfection. Next day, cells reached 90 to 95% confluence and were transfected with siRNA twice (4 h apart between first and second transfections) using Lipofectamine RNAiMAX (Invitrogen) according to the manufacturer’s protocol. Transfected NHDFs were incubated for 48 h and then infected with GFP-expressing TB40/E virus at an MOI of 3. GFP intensity was monitored every 24 h with a Synergy HT microplate reader (BioTek). The entire screen was performed in duplicate and repeated twice. At 7 days postinfection, 5 µl supernatant was transferred to fresh untransfected cells and GFP levels were monitored as described above.

### Measuring infectious virus production by plaque assay.

Following siRNA transfection and TB40/E infection, supernatant and cells were harvested at 7 dpi. Confluent NHDF cells were treated with a dilution series of either the supernatant or the cell contents and left for 12 h. A carboxymethyl cellulose overlay was added, and the formation of plaques was monitored. The assay was performed in duplicate on two biological replicates. Infectious progeny production was calculated, and the standard deviation between the biological replicates was determined.

### Cell viability assay.

NHDF cells were seeded and transfected as described for the siRNA screen above. Cell viability was assessed 48 h posttransfection by CellTiter-Blue (Promega), according to the manufacturer’s instructions. The assay was performed in duplicate and repeated twice. Results were normalized to negative-control-treated cells and expressed as a percentage of the siNeg levels (*n =* 2; error bars represent standard deviations between biological replicates).

### qRT-PCR.

Total RNA was harvested by Trizol purification, and following NanoDrop spectrophotometer analysis, 1 µg RNA was DNase treated (Promega) and reverse transcribed with a high-capacity cDNA RT kit (ABI). Gene-specific TaqMan primer-probe sets (ABI) were used to assess expression levels of COPA (Hs00189232_m1), COPB2 (Hs00178076_m1), ERC1 (Hs01553906_m1), and RAB4B (Hs01927479_s1). Custom primers were designed to assay samples for UL44, UL83, UL99, and UL94 RNA with the SensiFAST SYBR Hi-ROX kit from Bioline UK (BIO-92020), using the following primers: UL44 (pp52), 5′-CCGGGTCTGGATAACGATATTA and 5′-TTCTTGGTGTTAGGGACGAACT; UL83 (pp65), 5′-CAGGTACACCTTGACGTACTGG and 5′-AAAGAGCCCGACGTCTACTACA; UL94, 5′-AGACCGATTCCAGAACTTTGAA and 5′-AATACCATCGCACGTCATACTG; UL99 (pp28), 5′-TCTCTACGCTCCTACGACAACA and 5′-ATAATGGAGCTTTGCTGATGGT. qRT-PCR results were normalized to GAPDH (Hs02758991_g1) levels and then to siNeg levels at 7 dpi by the threshold cycle (ΔΔ*C*_*T*_) method. The assay was performed on two biological replicates, and error bars indicate the standard deviation between replicates. A two-tailed homoscedastic Student *t* test was applied to assess whether RNA levels after siCOPA treatment differed significantly from siNeg-treated cells (*P* > 0.05, not significant [NS]; *P* ≤ 0.05, *; *P* ≤ 0.01, **).

### qPCR.

DNA was purified using a DNeasy Blood and Tissue kit (Qiagen) and quantified with a NanoDrop spectrophotometer. The SensiFAST SYBR Hi-ROX kit from Bioline United Kingdom (BIO-92020) and custom gene-specific primer sets were used to assay 25 ng DNA per reaction for gB and GAPDH, using the following primers: GAPDH DNA, 5′-CCACTCCTGATTTCTGGAAAAG and 5′-GAAATTAACTGGACAGGGCAAG; UL55 (gB), 5′-TAGCTACGACGAAACGTCAAAA and 5′-GGTACGGATCTTATTCGCTTTG. Results were normalized to GAPDH DNA levels and then to siNeg levels at 1 dpi by the ΔΔ*C*_*T*_ method (*n =* 2; error bars represent standard deviations).

### Flow cytometry analysis.

NHDF cells were transfected as described previously (23) and infected with TB40/E GFP virus at MOIs of 0.1, 1, and 5 at 48 h posttransfection. Cells were harvested at 1 and 2 dpi, fixed in cold 1% paraformaldehyde (PFA) for 5 min, washed 3 times in ice-cold Dulbecco’s phosphate-buffered saline (DPBS), and assayed by FACSCalibur (BD Biosciences) for GFP signal. The mean fluorescence intensity (MFI) range of negative-control (uninfected) and positive-control (infected) cells was used to define the MFI range of GFP-negative and GFP-positive cells. The fold change of GFP-positive cells following siCOPA, siCOPB2, siERC1, and siRAB4B treatment was determined relative to GFP-positive cells from siNeg-treated wells at each MOI and time point.

### Western blot analysis.

Following transfection, cells were harvested daily at 1 to 7 dpi in RIPA buffer. Protein concentrations were determined by bicinchoninic acid (BCA) assay (Thermo Fisher) according to the manufacturer’s instructions. Proteins were separated on 4 to 12% Bis-Tris polyacrylamide gels (Invitrogen) and transferred to nitrocellulose membranes by either wet transfer (20% methanol [MeOH]) or Trans-Blot SD semidry transfer (mixed molecular weight). Membranes were probed with antibodies to viral proteins IE1 (Merck Millipore; MAB8131), IE2 (Merck Millipore; MAB8131), pp52 (Santa Cruz Biotechnology; sc-56971), and pp28 (Santa Cruz Biotechnology; sc-69749) and with antibodies to host proteins COPA (Santa Cruz Biotechnology; sc-398099), COPB2 (Novus Biologicals; NBP1-88651), ERC1 (Novus Biologicals; NBP1-88177), RAB4B (Santa Cruz Biotechnology; sc-26565), and actin (Abcam; ab8226). Secondary antibodies conjugated to IR800 dye (Li-Cor) were used, and blots were imaged by Li-Cor Odyssey infrared fluorescence. Quantification was done with Li-Cor Image Studio Lite software. Relative protein levels were calculated by first normalizing to the loading control (actin) and then by determining the ratio between the sample and its corresponding negative control.

### Immunofluorescence.

Normal human dermal fibroblasts (NHDFs) infected with laboratory-adapted HCMV strain AD169 were fixed and permeabilized in methanol-acetone solution (1:1) for 7 min and then blocked with 5% human serum in PBS for 30 min. Primary and secondary antibodies were diluted with 5% human serum in PBS. Cells were washed with PBS after primary and after secondary antibody incubations. Primary antibodies were mouse anti-HCMV pp28 monoclonal antibody (CH19; Santa Cruz Biotechnology) at 1:500 and rabbit anti-Rab4B polyclonal antibody (C-3; Santa Cruz Biotechnology) at 1:500. Alexa Fluor 647-conjugated goat anti-mouse and Alexa Fluor 488-conjugated goat anti-rabbit IgG secondary antibodies (Invitrogen) were diluted 1:1,000. All images were acquired with a Zeiss LSM 710 confocal microscope fitted with a 63×/1.4 oil-immersion objective lens.

### Virion DNase protection assay.

For supernatant viral genomes, virus was isolated by ultracentrifugation over a sorbitol cushion as previously described (39). The viral pellet was resuspended in DNase buffer and split into two aliquots, one of which was treated for 1 h at 37°C with Turbo DNase (Ambion). DNA was then isolated by phenol-chloroform purification, and PCR analysis was performed as described above.

### Bioinformatics and statistical analysis.

siRNA screen data were analyzed using Excel (Microsoft) and Prism 6.0 (GraphPad). Log_2_ transformations and correlations were performed with Prism 6.0, and Spearman’s rank-order correlation was used to assess the significance of correlations. Two-tailed homoscedastic Student’s *t* test was applied to assess whether primary replication and virus production results differed significantly (*P* > 0.05, NS; *P* ≤ 0.05, *; *P* ≤ 0.01, **; *P* ≤ 0.001, ***; *P* ≤ 0.0001, ****).
