# Supplementary figures and images for: Identification of Host Factors Involved in Human Cytomegalovirus Replication, Assembly, and Egress Using a Two-Step Small Interfering RNA Screen
Source: mBio. 2018 Jun 26;9(3):e00716-18. doi: 10.1128/mBio.00716-18 (PMC6020295; doi:10.1128/mBio.00716-18)

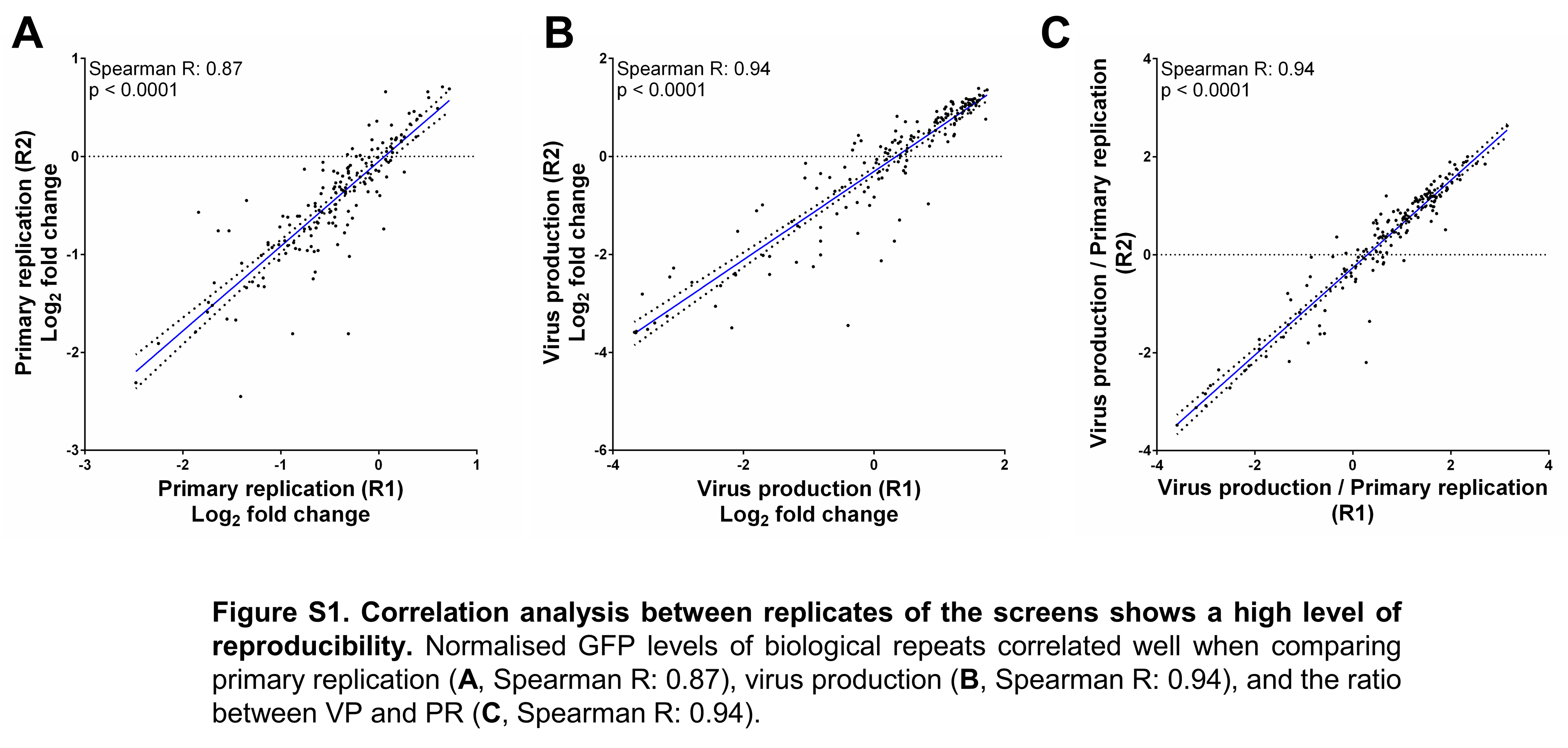

Supplement: FIG S1 [file mbo003183951sf1.tif]

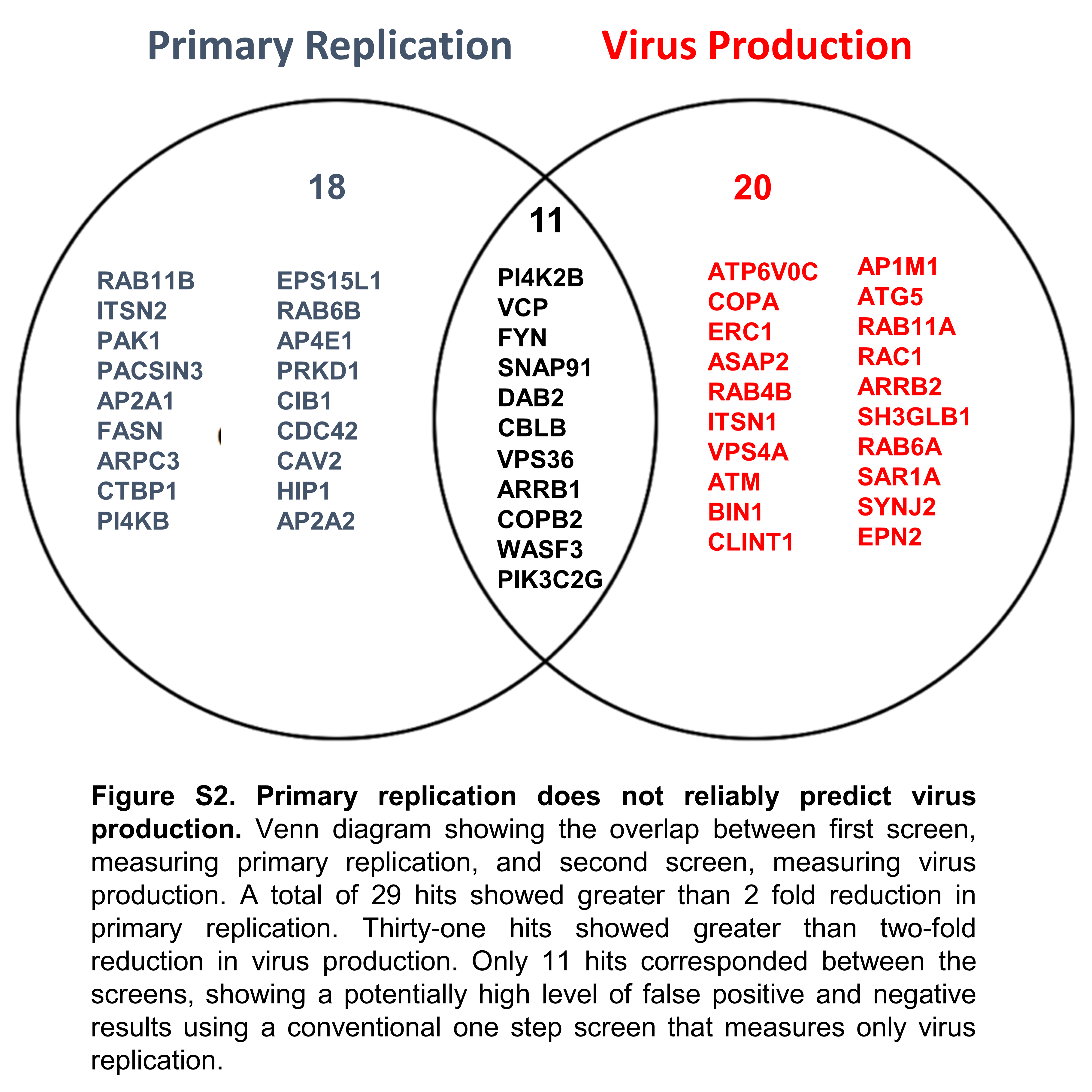

Supplement: FIG S2 [file mbo003183951sf2.tif]

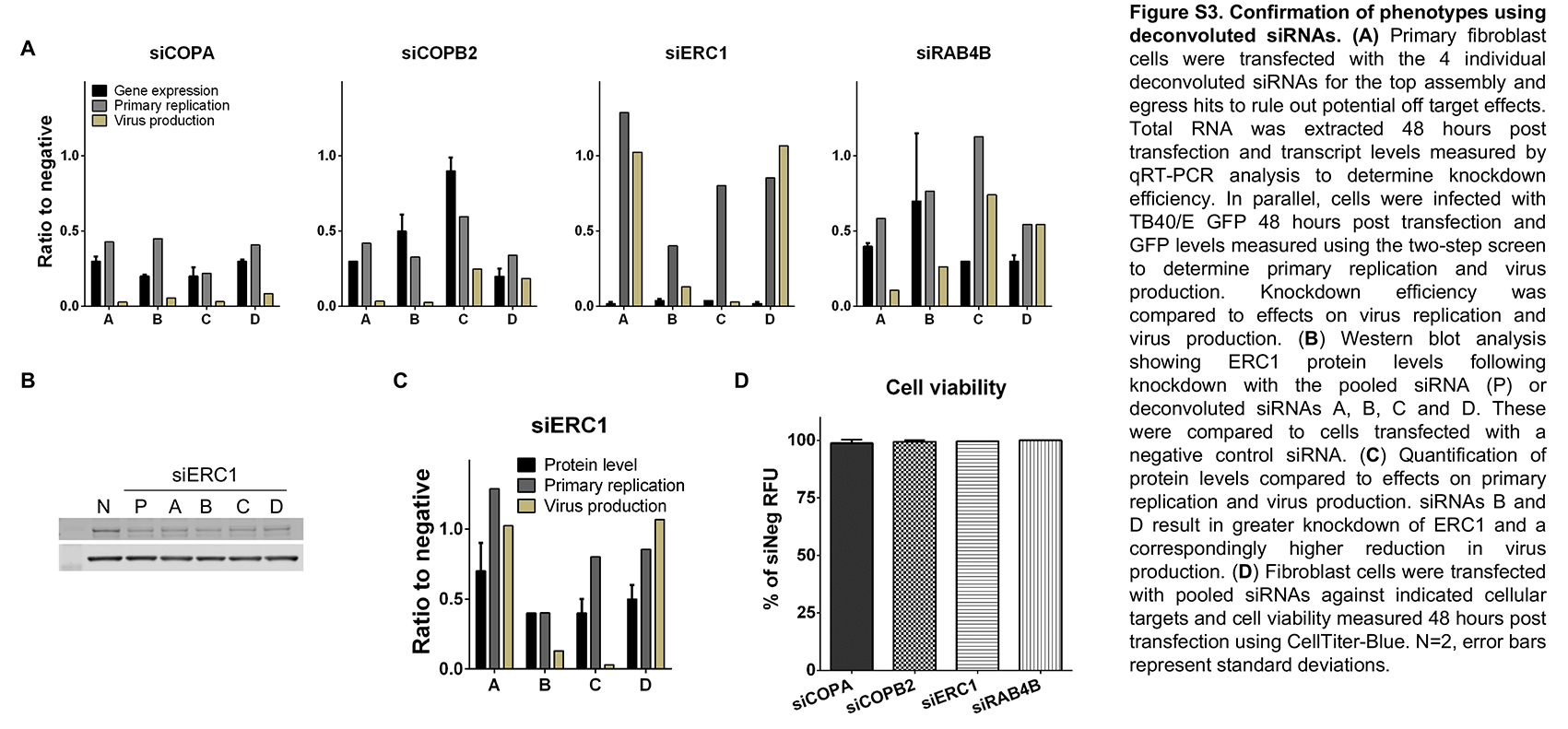

Supplement: FIG S3 [file mbo003183951sf3.jpg]

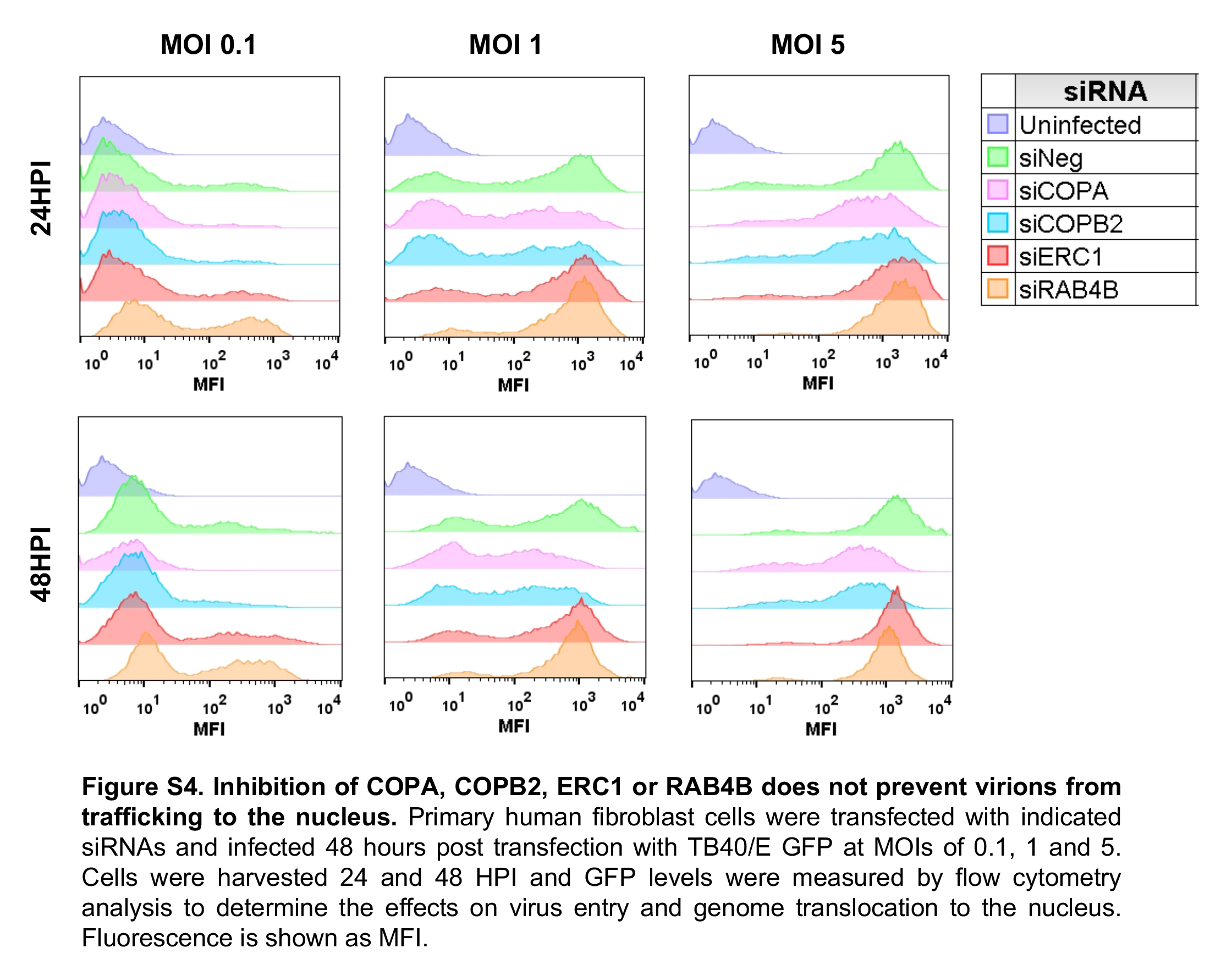

Supplement: FIG S4 [file mbo003183951sf4.jpg]

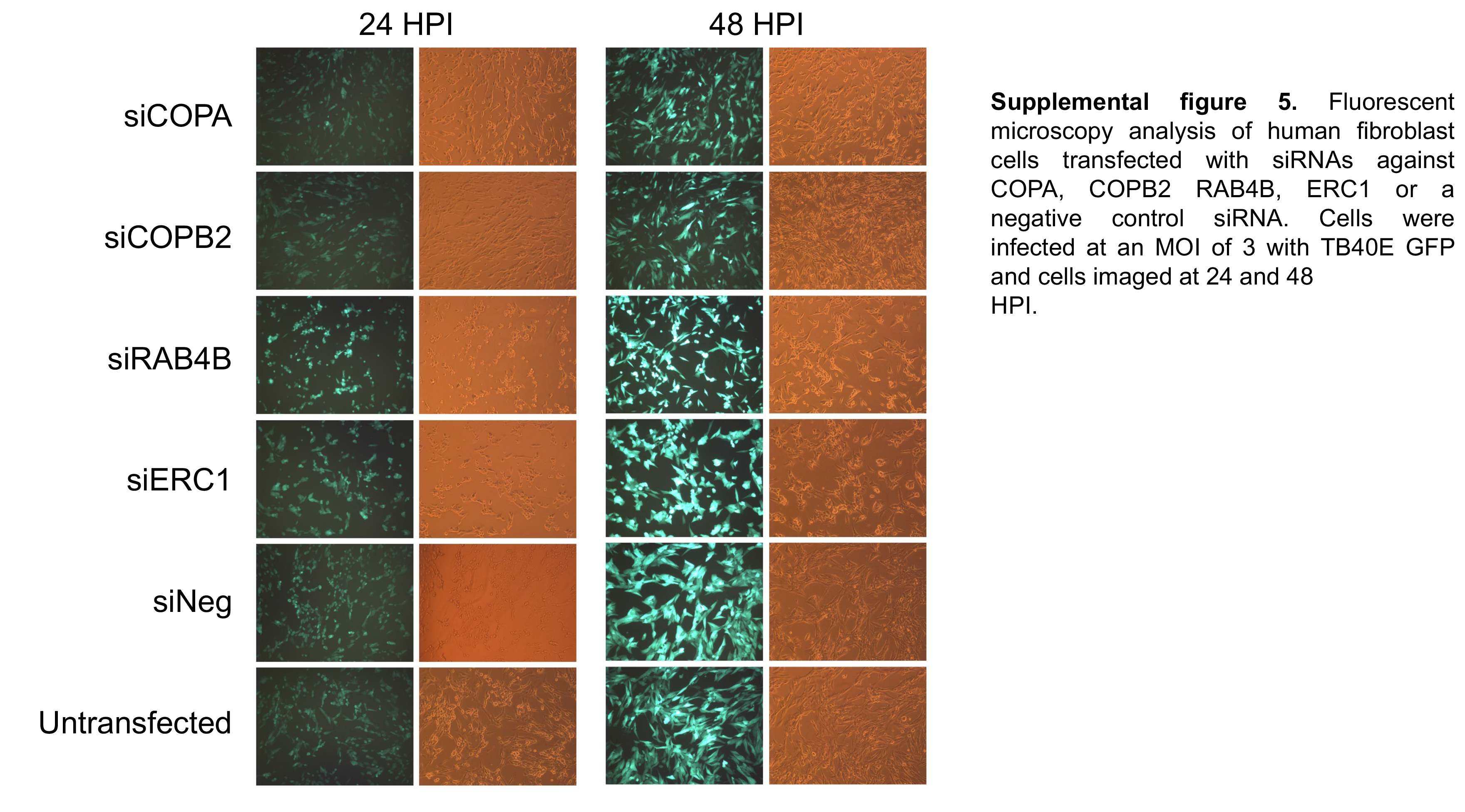

Supplement: FIG S5 [file mbo003183951sf5.jpg]

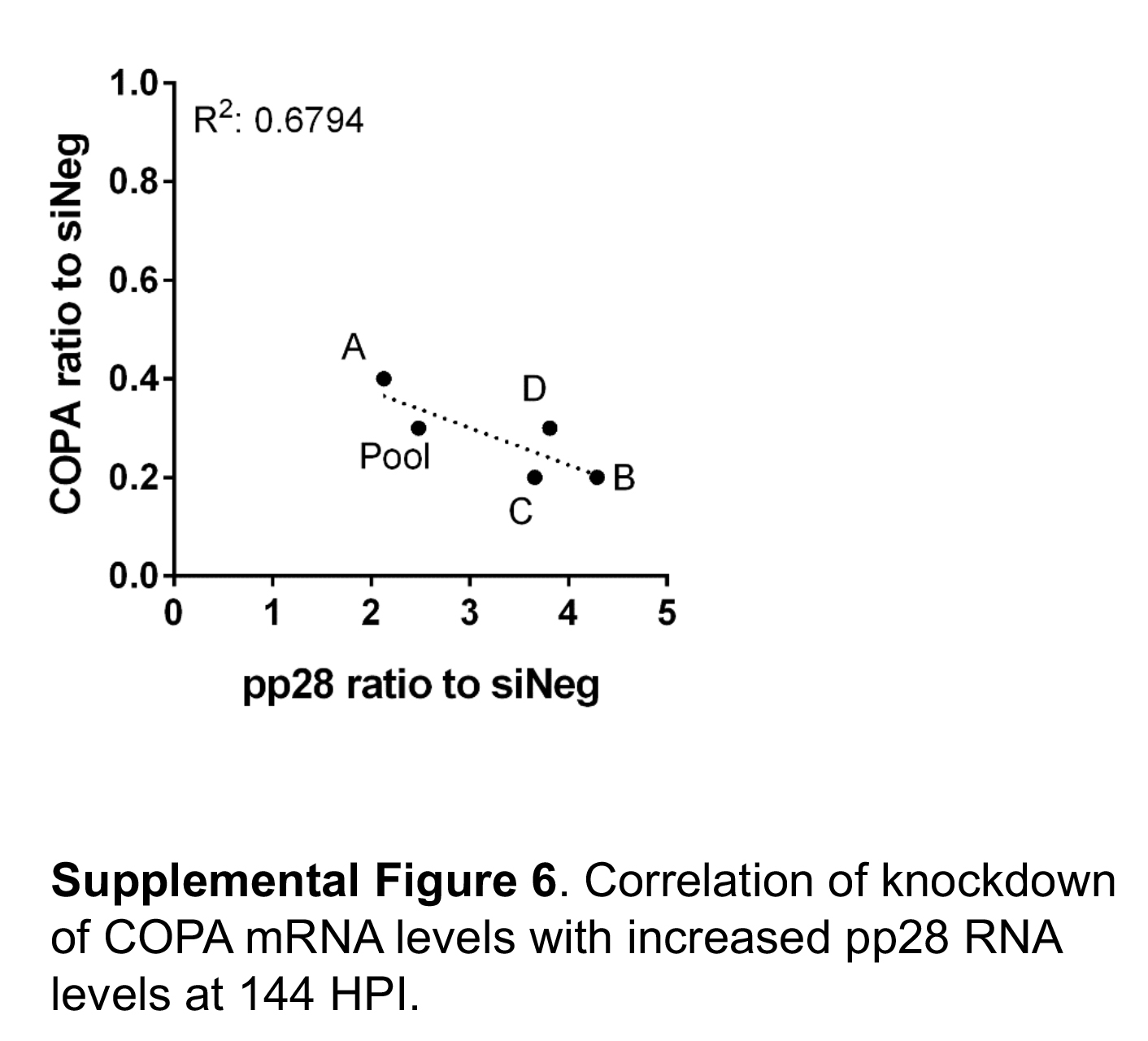

Supplement: FIG S6 [file mbo003183951sf6.jpg]

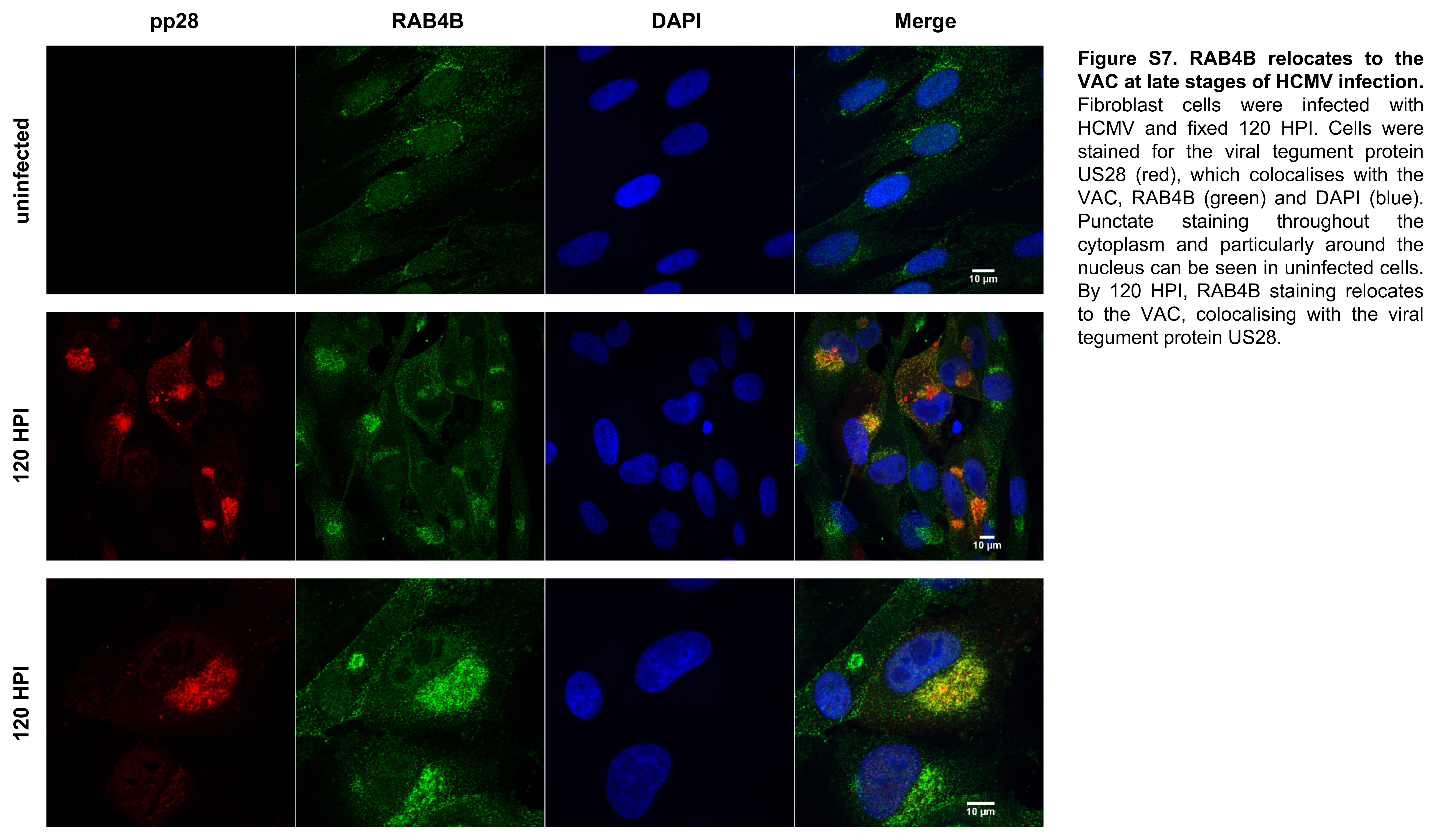

Supplement: FIG S7 [file mbo003183951sf7.jpg]
