# Supplementary material for: Identification of Host Factors Involved in Human Cytomegalovirus Replication, Assembly, and Egress Using a Two-Step Small Interfering RNA Screen
Source: mBio. 2018 Jun 26;9(3):e00716-18. doi: 10.1128/mBio.00716-18 (PMC6020295; doi:10.1128/mBio.00716-18)
Supplement: TABLE S1 [file mbo003183951st1.pdf]

A

Gene	PR Fold change	PR Std dev	Description
PI4K2B	-5.3	0.45	Phosphatidylinositol 4-kinase type 2 beta
VCP	-4.3	0.70	Valosin containing protein
FYN	-4.1	1.98	FYN proto-oncogene, Src family tyrosine kinase
SNAP91	-3.6	0.13	Synaptosomal-associated protein, 91kDa
RAB11B	-3.2	0.24	RAB11B, member RAS oncogene family
DAB2	-3.1	0.38	Dab, mitogen-responsive phosphoprotein, homolog 2 (Drosophila)
ITSN2	-3.1	0.27	Intersectin 2
CBLB	-3.1	0.17	Cbl proto-oncogene B, E3 ubiquitin protein ligase
VPS36	-3.0	0.30	Vacuolar protein sorting 36 homolog (S. cerevisiae)
PAK1	-2.8	0.55	p21 protein (Cdc42/Rac)-activated kinase 1
PACSIN3	-2.7	1.18	Protein kinase C and casein kinase substrate in neurons 3
AP2A1	-2.5	0.00	Adaptor-related protein complex 2, alpha 1 subunit
FASN	-2.5	1.49	Fatty acid synthase
ARPC3	-2.4	0.02	Actin related protein 2/3 complex, subunit 3, 21kDa
ARRB1	-2.4	0.10	Arrestin, beta 1
CTBP1	-2.4	1.01	C-terminal binding protein 1
PI4KB	-2.4	0.36	Phosphatidylinositol 4-kinase, catalytic, beta
EPS15L1	-2.4	0.19	Epidermal growth factor receptor pathway substrate 15-like 1
RAB6B	-2.4	1.60	RAB6B, member RAS oncogene family
AP4E1	-2.3	0.06	Adaptor-related protein complex 4, epsilon 1 subunit
PRKD1	-2.3	0.84	Protein kinase D1
CIB1	-2.2	0.20	Calcium and integrin binding 1 (calmyrin)
CDC42	-2.1	0.04	Cell division cycle 42
CAV2	-2.0	0.23	Caveolin 2
HIP1	-2.0	0.16	Huntingtin interacting protein 1
COPB2	-2.0	0.25	Coatomer protein complex, subunit beta 2 (beta prime)
WASF3	-2.0	0.55	WAS protein family, member 3
AP2A2	-2.0	0.04	Adaptor-related protein complex 2, alpha 2 subunit
PIK3C2G	-2.0	0.83	Phosphatidylinositol-4-phosphate 3-kinase, catalytic subunit type 2 gamma
PITPNM1	-1.9	0.12	Phosphatidylinositol transfer protein, membrane-associated 1
MAPK8IP2	-1.9	0.49	Mitogen-activated protein kinase 8 interacting protein 2
ARF3	-1.9	0.03	ADP-ribosylation factor 3
AP1B1	-1.9	0.09	Adaptor-related protein complex 1, beta 1 subunit
SH3GLB1	-1.9	0.08	SH3-domain GRB2-like endophilin B1
CLINT1	-1.8	0.13	Clathrin interactor 1
GNB2L1	-1.8	0.01	Guanine nucleotide binding protein (G protein), beta polypeptide 2-like 1
ROCK2	-1.8	0.27	Rho-associated, coiled-coil containing protein kinase 2
ATP6V0A1	-1.8	0.20	ATPase, H+ transporting, lysosomal V0 subunit a1
ITSN1	-1.8	0.27	Intersectin 1 (SH3 domain protein)
RAB5B	-1.8	0.20	RAB5B, member RAS oncogene family
CLTA	-1.8	0.17	Clathrin, light chain A
CBL	-1.7	0.32	Cbl proto-oncogene, E3 ubiquitin protein ligase
VAMP1	-1.7	0.29	Vesicle-associated membrane protein 1 (synaptobrevin 1)
TNIK	-1.7	0.26	TRAF2 and NCK interacting kinase
RAB1A	-1.7	0.02	RAB1A, member RAS oncogene family
WASF2	-1.7	0.10	WAS protein family, member 2
HGS	-1.7	0.03	Hepatocyte growth factor-regulated tyrosine kinase substrate
IP6K3	-1.7	0.18	Inositol hexakisphosphate kinase 3
ATG5	-1.6	0.16	Autophagy related 5
EPN2	-1.6	0.57	Epsin 2
COPA	-1.6	0.07	Coatomer protein complex, subunit alpha
ASAP2	-1.6	0.37	ArfGAP with SH3 domain, ankyrin repeat and PH domain 2
PIP5K1A	-1.6	0.03	Phosphatidylinositol-4-phosphate 5-kinase, type I, alpha
DNM1	-1.6	0.04	Dynamin 1
EPN1	-1.5	0.16	Epsin 1
MAP1LC3A	-1.5	0.12	Microtubule-associated protein 1 light chain 3 alpha
ATM	-1.5	0.20	ATM serine/threonine kinase
RAB11A	-1.5	0.04	RAB11A, member RAS oncogene family
ATP6V0C	-1.5	0.10	ATPase, H+ transporting, lysosomal 16kDa, V0 subunit c
HIP1R	-1.5	0.31	Huntingtin interacting protein 1 related
PDCD6IP	-1.5	0.15	Programmed cell death 6 interacting protein
DIAPH1	-1.5	0.01	Diaphanous-related formin 1
VAV2	-1.5	0.12	VAV 2 guanine nucleotide exchange factor
WAS	-1.5	0.04	Wiskott-Aldrich syndrome
ARPC1B	-1.4	0.12	Actin related protein 2/3 complex, subunit 1B, 41kDa
VAPB	-1.4	0.05	VAMP (vesicle-associated membrane protein)-associated protein B and C
EZR	-1.4	0.27	Ezrin
SYNJ1	-1.4	0.14	Synaptojanin 1
MAP4K2	-1.4	0.07	Mitogen-activated protein kinase kinase kinase kinase 2
CFL1	-1.4	0.42	Cofilin 1 (non-muscle)
RAB2A	-1.4	0.04	RAB2A, member RAS oncogene family
RAC1	-1.4	0.24	RAS-related C3 botulinum toxin substrate 1 (Rho family, small GTP binding protein Rac1)
ARF6	-1.4	0.16	ADP-ribosylation factor 6
EFS	-1.4	0.16	Embryonal Fyn-associated substrate
RAB5C	-1.3	0.05	RAB5C, member RAS oncogene family
ACACA	-1.3	0.09	Acetyl-CoA carboxylase alpha
AP2M1	-1.3	0.09	Adaptor-related protein complex 2, mu 1 subunit
CIB2	-1.3	0.27	Calcium and integrin binding family member 2
RAB8B	-1.3	0.17	RAB8B, member RAS oncogene family
CBLC	-1.3	0.03	Cbl proto-oncogene C, E3 ubiquitin protein ligase
ARF1	-1.3	0.02	ADP-ribosylation factor 1
CAMK1	-1.3	0.06	Calcium/calmodulin-dependent protein kinase I
VAMP2	-1.3	0.11	Vesicle-associated membrane protein 2 (synaptobrevin 2)
PI4KA	-1.3	0.01	Phosphatidylinositol 4-kinase, catalytic, alpha
EPN3	-1.3	0.01	Epsin 3
SREBF1	-1.3	0.31	Sterol regulatory element binding transcription factor 1
RAB4A	-1.3	0.08	RAB4A, member RAS oncogene family
PIK3CG	-1.3	0.01	Phosphatidylinositol-4,5-bisphosphate 3-kinase, catalytic subunit gamma
NEDD4	-1.2	0.19	Neural precursor cell expressed, developmentally down-regulated 4, E3 ubiquitin protein ligase
MAPK8IP1	-1.2	0.15	Mitogen-activated protein kinase 8 interacting protein 1
CAV3	-1.2	0.06	Caveolin 3
ARF5	-1.2	0.10	ADP-ribosylation factor 5
SYNJ2	-1.2	0.04	Synaptojanin 2
AP1M1	-1.2	0.04	Adaptor-related protein complex 1, mu 1 subunit
RAB3B	-1.2	0.08	RAB3B, member RAS oncogene family
SH3GLB2	-1.2	0.15	SH3-domain GRB2-like endophilin B2
CIB3	-1.2	0.09	Calcium and integrin binding family member 3
BECN1	-1.2	0.11	Beclin 1, autophagy related
SNX1	-1.2	0.12	Sorting nexin 1
RAB6A	-1.2	0.08	RAB6A, member RAS oncogene family
RAB8A	-1.2	0.06	RAB8A, member RAS oncogene family
ADAM10	-1.2	0.19	ADAM metallopeptidase domain 10
CAV1	-1.1	0.01	Caveolin 1, caveolae protein, 22kDa
VAPA	-1.1	0.12	VAMP (vesicle-associated membrane protein)-associated protein A, 33kDa
GRB2	-1.1	0.03	Growth factor receptor-bound protein 2
SAR1A	-1.1	0.09	Secretion associated, RAS related GTPase 1A
SEC13	-1.1	0.07	SEC13 homolog (S. cerevisiae)
PACSIN1	-1.1	0.03	Protein kinase C and casein kinase substrate in neurons 1
SYT2	-1.1	0.12	Synaptotagmin II
ACTR3	-1.1	0.07	ARP3 actin-related protein 3 homolog (yeast)
RAB5A	-1.1	0.03	RAB5A, member RAS oncogene family
EEA1	-1.1	0.06	Early endosome antigen 1
RAB29	-1.1	0.01	RAB29, member RAS oncogene family
BIN1	-1.1	0.08	Bridging integrator 1
ARPC4	-1.0	0.05	Actin related protein 2/3 complex, subunit 4, 20kDa
ARPC5	-1.0	0.00	Actin related protein 2/3 complex, subunit 5, 16kDa
WASF1	-0.3	1.91	WAS protein family, member 1
DNM2	-0.2	1.65	Dynamin 2
ACTR2	-0.1	1.60	ARP2 actin-related protein 2 homolog (yeast)
CD81	-0.1	1.60	CD81 molecule
CLTB	-0.1	1.52	Clathrin, light chain B
ARRB2	0.0	1.57	Arrestin, beta 2
NSF	0.0	1.48	N-ethylmaleimide-sensitive factor
CLTC	0.0	1.47	Clathrin, heavy chain (Hc)
ATG12	0.0	1.49	Autophagy related 12
AP3D1	0.0	1.46	Adaptor-related protein complex 3, delta 1 subunit
ERC1	0.0	1.50	ELKS/RAB6-interacting/CAST family member 1
MAPK8IP3	0.0	1.54	Mitogen-activated protein kinase 8 interacting protein 3
PI4K2A	0.0	1.64	Phosphatidylinositol 4-kinase type 2 alpha
SNX2	0.1	1.64	Sorting nexin 2
EPS15	0.1	1.62	Epidermal growth factor receptor pathway substrate 15
AP1M2	1.0	0.02	Adaptor-related protein complex 1, mu 2 subunit
AMPH	1.1	0.05	Amphiphysin
LIMK1	1.1	0.05	LIM domain kinase 1
DNM3	1.1	0.03	Dynamin 3
VPS4A	1.1	0.01	Vacuolar protein sorting 4 homolog A (S. cerevisiae)
ARFIP2	1.1	0.03	ADP-ribosylation factor interacting protein 2
ARPC2	1.1	0.01	Actin related protein 2/3 complex, subunit 2, 34kDa
GORASP1	1.1	0.00	Golgi reassembly stacking protein 1, 65kDa
RAB3A	1.1	0.04	RAB3A, member RAS oncogene family
SYT1	1.1	0.04	Synaptotagmin I
STAU1	1.2	0.01	Staufen double-stranded RNA binding protein 1
CYTH3	1.2	0.11	Cytohesin 3
RAB11FIP5	1.2	0.04	RAB11 family interacting protein 5 (class I)
TSG101	1.2	0.10	Tumor susceptibility 101
RAB7B	1.3	0.01	RAB7B, member RAS oncogene family
GIT1	1.3	0.08	G protein-coupled receptor kinase interacting ArfGAP 1
NEDD4L	1.3	0.01	Neural precursor cell expressed, developmentally down-regulated 4-like, E3 ubiquitin protein ligase
RAB4B	1.3	0.37	RAB4B, member RAS oncogene family
RHOA	1.3	0.06	RAS homolog family member A
RAB3D	1.3	0.00	RAB3D, member RAS oncogene family
PICALM	1.5	0.07	Phosphatidylinositol binding clathrin assembly protein
CLTCL1	1.5	0.07	Clathrin, heavy chain-like 1
RAB3C	1.5	0.11	RAB3C, member RAS oncogene family
AP2B1	1.6	0.05	Adaptor-related protein complex 2, beta 1 subunit
ROCK1	1.6	0.02	Rho-associated, coiled-coil containing protein kinase 1

B

Gene	PR Fold change	PR Std dev	VP Fold change	VP Std dev	Description
VCP	-4.3	0.70	-12.4	0.49	Valosin containing protein
COPB2	-2.0	0.25	-12.3	0.30	Coatomer protein complex, subunit beta 2 (beta prime)
ATP6V0C	-1.5	0.10	-12.3	0.34	ATPase, H+ transporting, lysosomal 16kDa, V0 subunit c
COPA	-1.6	0.07	-12.2	0.44	Coatomer protein complex, subunit alpha
ERC1	0.0	1.50	-11.3	0.30	ELKS/RAB6-interacting/CAST family member 1
VPS36	-3.0	0.30	-10.4	0.18	Vacuolar protein sorting 36 homolog (S. cerevisiae)
PIK3C2G	-2.0	0.83	-9.4	3.34	Phosphatidylinositol-4-phosphate 3-kinase, catalytic subunit type 2 gamma
ASAP2	-1.6	0.37	-9.3	0.42	ArfGAP with SH3 domain, ankyrin repeat and PH domain 2
FYN	-4.1	1.98	-7.9	4.79	FYN proto-oncogene, Src family tyrosine kinase
RAB4B	1.3	0.37	-7.1	2.29	RAB4B, member RAS oncogene family
ITSN1	-1.8	0.27	-6.9	2.09	Intersectin 1 (SH3 domain protein)
DAB2	-3.1	0.38	-6.6	2.48	Dab, mitogen-responsive phosphoprotein, homolog 2 (Drosophila)
VPS4A	1.1	0.01	-6.4	0.71	Vacuolar protein sorting 4 homolog A (S. cerevisiae)
WASF3	-2.0	0.55	-6.1	6.81	WAS protein family, member 3
ATM	-1.5	0.20	-5.7	0.82	ATM serine/threonine kinase
BIN1	-1.1	0.08	-4.9	0.72	Bridging integrator 1
CLINT1	-1.8	0.13	-4.8	0.60	Clathrin interactor 1
AP1M1	-1.2	0.04	-4.2	1.61	Adaptor-related protein complex 1, mu 1 subunit
ARRB1	-2.4	0.10	-3.6	0.58	Arrestin, beta 1
ATG5	-1.6	0.16	-3.6	0.96	Autophagy related 5
CBLB	-3.1	0.17	-3.6	0.76	Cbl proto-oncogene B, E3 ubiquitin protein ligase
RAB11A	-1.5	0.04	-3.4	1.55	RAB11A, member RAS oncogene family
RAC1	-1.4	0.24	-3.3	2.01	RAS-related C3 botulinum toxin substrate 1 (Rho family, small GTP binding protein Rac1)
ARRB2	0.0	1.57	-2.9	1.60	Arrestin, beta 2
PI4K2B	-5.3	0.45	-2.8	0.94	Phosphatidylinositol 4-kinase type 2 beta
SH3GLB1	-1.9	0.08	-2.6	0.91	SH3-domain GRB2-like endophilin B1
RAB6A	-1.2	0.08	-2.5	1.09	RAB6A, member RAS oncogene family
SAR1A	-1.1	0.09	-2.5	0.31	Secretion associated, RAS related GTPase 1A
SNAP91	-3.6	0.13	-2.5	0.08	Synaptosomal-associated protein, 91kDa
SYNJ2	-1.2	0.04	-2.2	0.69	Synaptojanin 2
EPN2	-1.6	0.57	-2.1	1.26	Epsin 2
PIP5K1A	-1.6	0.03	-1.9	0.54	Phosphatidylinositol-4-phosphate 5-kinase, type I, alpha
CLTB	-0.1	1.52	-1.7	0.51	Clathrin, light chain B
ARF3	-1.9	0.03	-1.7	0.50	ADP-ribosylation factor 3
AP2A1	-2.5	0.00	-1.7	0.19	Adaptor-related protein complex 2, alpha 1 subunit
RAB6B	-2.4	1.60	-1.7	3.86	RAB6B, member RAS oncogene family
EPN1	-1.5	0.16	-1.6	0.68	Epsin 1
RAB5C	-1.3	0.05	-1.6	0.08	RAB5C, member RAS oncogene family
GNB2L1	-1.8	0.01	-1.6	0.51	Guanine nucleotide binding protein (G protein), beta polypeptide 2-like 1
WASF2	-1.7	0.10	-1.5	0.67	WAS protein family, member 2
CAMK1	-1.3	0.06	-1.5	0.34	Calcium/calmodulin-dependent protein kinase I
RAB1A	-1.7	0.02	-1.5	0.10	RAB1A, member RAS oncogene family
RAB11FIP5	1.2	0.04	-1.5	0.18	RAB11 family interacting protein 5 (class I)
CLTC	0.0	1.47	-1.4	0.38	Clathrin, heavy chain (Hc)
RAB8B	-1.3	0.17	-1.3	0.07	RAB8B, member RAS oncogene family
EFS	-1.4	0.16	-1.3	0.34	Embryonal Fyn-associated substrate
CD81	-0.1	1.60	-1.3	0.15	CD81 molecule
ARF5	-1.2	0.10	-1.1	0.01	ADP-ribosylation factor 5
ADAM10	-1.2	0.19	-1.0	3.22	ADAM metallopeptidase domain 10
MAPK8IP2	-1.9	0.49	-0.6	2.67	Mitogen-activated protein kinase 8 interacting protein 2
DNM1	-1.6	0.04	-0.4	1.97	Dynamin 1
ARF1	-1.3	0.02	-0.2	1.71	ADP-ribosylation factor 1
RAB2A	-1.4	0.04	-0.1	1.78	RAB2A, member RAS oncogene family
RAB3A	1.1	0.04	-0.1	1.63	RAB3A, member RAS oncogene family
PACSIN3	-2.7	1.18	-0.1	2.65	Protein kinase C and casein kinase substrate in neurons 3
CLTA	-1.8	0.17	-0.1	1.66	Clathrin, light chain A
VAV2	-1.5	0.12	0.0	1.49	VAV 2 guanine nucleotide exchange factor
SYT1	1.1	0.04	0.0	1.67	Synaptotagmin I
CFL1	-1.4	0.42	0.0	1.73	Cofilin 1 (non-muscle)
FASN	-2.5	1.49	0.0	1.47	Fatty acid synthase
ARFIP2	1.1	0.03	0.0	1.54	ADP-ribosylation factor interacting protein 2
PRKD1	-2.3	0.84	0.1	1.70	Protein kinase D1
EPS15L1	-2.4	0.19	0.1	1.88	Epidermal growth factor receptor pathway substrate 15-like 1
HIP1R	-1.5	0.31	0.1	1.83	Huntingtin interacting protein 1 related
CTBP1	-2.4	1.01	0.1	1.79	C-terminal binding protein 1
PACSIN1	-1.1	0.03	0.1	1.68	Protein kinase C and casein kinase substrate in neurons 1
CAV2	-2.0	0.23	0.1	1.80	Caveolin 2
ARPC3	-2.4	0.02	0.2	1.75	Actin related protein 2/3 complex, subunit 3, 21kDa
AP2M1	-1.3	0.09	1.1	0.05	Adaptor-related protein complex 2, mu 1 subunit
CIB2	-1.3	0.27	1.1	0.08	Calcium and integrin binding family member 2
CBL	-1.7	0.32	1.1	0.05	Cbl proto-oncogene, E3 ubiquitin protein ligase
HIP1	-2.0	0.16	1.1	0.01	Huntingtin interacting protein 1
CIB1	-2.2	0.20	1.1	0.06	Calcium and integrin binding 1 (calmyrin)
AP4E1	-2.3	0.06	1.2	0.21	Adaptor-related protein complex 4, epsilon 1 subunit
GORASP1	1.1	0.00	1.2	0.00	Golgi reassembly stacking protein 1, 65kDa
EPN3	-1.3	0.01	1.2	0.24	Epsin 3
RAB8A	-1.2	0.06	1.2	0.06	RAB8A, member RAS oncogene family
CBLC	-1.3	0.03	1.3	0.01	Cbl proto-oncogene C, E3 ubiquitin protein ligase
SREBF1	-1.3	0.31	1.3	0.06	Sterol regulatory element binding transcription factor 1
PDCD6IP	-1.5	0.15	1.3	0.21	Programmed cell death 6 interacting protein
GIT1	1.3	0.08	1.3	0.02	G protein-coupled receptor kinase interacting ArfGAP 1
SYNJ1	-1.4	0.14	1.3	0.13	Synaptojanin 1
AP2A2	-2.0	0.04	1.4	0.14	Adaptor-related protein complex 2, alpha 2 subunit
HGS	-1.7	0.03	1.4	0.11	Hepatocyte growth factor-regulated tyrosine kinase substrate
ITSN2	-3.1	0.27	1.4	0.15	Intersectin 2
ARPC2	1.1	0.01	1.5	0.46	Actin related protein 2/3 complex, subunit 2, 34kDa
SNX1	-1.2	0.12	1.5	0.12	Sorting nexin 1
AP3D1	0.0	1.46	1.5	0.18	Adaptor-related protein complex 3, delta 1 subunit
MAPK8IP3	0.0	1.54	1.5	0.35	Mitogen-activated protein kinase 8 interacting protein 3
CAV3	-1.2	0.06	1.6	0.43	Caveolin 3
DNM2	-0.2	1.65	1.6	0.28	Dynamin 2
PAK1	-2.8	0.55	1.6	0.04	p21 protein (Cdc42/Rac)-activated kinase 1
RAB5A	-1.1	0.03	1.6	0.25	RAB5A, member RAS oncogene family
LIMK1	1.1	0.05	1.7	0.34	LIM domain kinase 1
MAP1LC3A	-1.5	0.12	1.7	0.68	Microtubule-associated protein 1 light chain 3 alpha
ARPC5	-1.0	0.00	1.7	0.54	Actin related protein 2/3 complex, subunit 5, 16kDa
CAV1	-1.1	0.01	1.8	0.20	Caveolin 1, caveolae protein, 22kDa
TNIK	-1.7	0.26	1.8	0.25	TRAF2 and NCK interacting kinase
NEDD4L	1.3	0.01	1.8	0.19	Neural precursor cell expressed, developmentally down-regulated 4-like, E3 ubiquitin protein ligase
PITPNM1	-1.9	0.12	1.8	0.17	Phosphatidylinositol transfer protein, membrane-associated 1
EZR	-1.4	0.27	1.8	0.69	Ezrin
IP6K3	-1.7	0.18	1.8	0.17	Inositol hexakisphosphate kinase 3
RAB5B	-1.8	0.20	1.9	0.69	RAB5B, member RAS oncogene family
ARPC4	-1.0	0.05	1.9	0.27	Actin related protein 2/3 complex, subunit 4, 20kDa
VAPB	-1.4	0.05	1.9	0.30	VAMP (vesicle-associated membrane protein)-associated protein B and C
NSF	0.0	1.48	1.9	0.22	N-ethylmaleimide-sensitive factor
STAU1	1.2	0.01	1.9	0.04	Staufen double-stranded RNA binding protein 1
AP1M2	1.0	0.02	1.9	0.30	Adaptor-related protein complex 1, mu 2 subunit
MAP4K2	-1.4	0.07	2.0	0.55	Mitogen-activated protein kinase kinase kinase kinase 2
AP1B1	-1.9	0.09	2.0	0.54	Adaptor-related protein complex 1, beta 1 subunit
WAS	-1.5	0.04	2.0	0.22	Wiskott-Aldrich syndrome
PICALM	1.5	0.07	2.0	0.29	Phosphatidylinositol binding clathrin assembly protein
ACACA	-1.3	0.09	2.0	0.26	Acetyl-CoA carboxylase alpha
RAB4A	-1.3	0.08	2.0	0.11	RAB4A, member RAS oncogene family
SEC13	-1.1	0.07	2.0	0.48	SEC13 homolog (S. cerevisiae)
CDC42	-2.1	0.04	2.1	0.43	Cell division cycle 42
NEDD4	-1.2	0.19	2.1	0.09	Neural precursor cell expressed, developmentally down-regulated 4, E3 ubiquitin protein ligase
PI4KB	-2.4	0.36	2.1	0.55	Phosphatidylinositol 4-kinase, catalytic, beta
PI4KA	-1.3	0.01	2.1	0.27	Phosphatidylinositol 4-kinase, catalytic, alpha
CLTCL1	1.5	0.07	2.1	0.22	Clathrin, heavy chain-like 1
BECN1	-1.2	0.11	2.1	0.41	Beclin 1, autophagy related
ATP6V0A1	-1.8	0.20	2.2	0.50	ATPase, H+ transporting, lysosomal V0 subunit a1
RAB3B	-1.2	0.08	2.2	0.16	RAB3B, member RAS oncogene family
SNX2	0.1	1.64	2.2	0.07	Sorting nexin 2
VAPA	-1.1	0.12	2.2	0.10	VAMP (vesicle-associated membrane protein)-associated protein A, 33kDa
VAMP1	-1.7	0.29	2.2	0.48	Vesicle-associated membrane protein 1 (synaptobrevin 1)
EEA1	-1.1	0.06	2.3	0.40	Early endosome antigen 1
PIK3CG	-1.3	0.01	2.3	0.37	Phosphatidylinositol-4,5-bisphosphate 3-kinase, catalytic subunit gamma
PI4K2A	0.0	1.64	2.3	0.66	Phosphatidylinositol 4-kinase type 2 alpha
CIB3	-1.2	0.09	2.3	0.43	Calcium and integrin binding family member 3
ARPC1B	-1.4	0.12	2.3	0.56	Actin related protein 2/3 complex, subunit 1B, 41kDa
RAB11B	-3.2	0.24	2.3	0.41	RAB11B, member RAS oncogene family
RAB7B	1.3	0.01	2.4	0.29	RAB7B, member RAS oncogene family
ACTR2	-0.1	1.60	2.4	0.28	ARP2 actin-related protein 2 homolog (yeast)
ATG12	0.0	1.49	2.4	0.38	Autophagy related 12
TSG101	1.2	0.10	2.4	0.47	Tumor susceptibility 101
CYTH3	1.2	0.11	2.4	0.46	Cytohesin 3
ACTR3	-1.1	0.07	2.4	0.53	ARP3 actin-related protein 3 homolog (yeast)
SYT2	-1.1	0.12	2.5	0.27	Synaptotagmin II
RHOA	1.3	0.06	2.5	0.35	RAS homolog family member A
WASF1	-0.3	1.91	2.5	1.12	WAS protein family, member 1
ARF6	-1.4	0.16	2.5	0.36	ADP-ribosylation factor 6
DNM3	1.1	0.03	2.5	0.63	Dynamin 3
GRB2	-1.1	0.03	2.5	0.61	Growth factor receptor-bound protein 2
RAB3D	1.3	0.00	2.5	0.39	RAB3D, member RAS oncogene family
AMPH	1.1	0.05	2.5	0.50	Amphiphysin
ROCK2	-1.8	0.27	2.5	0.45	Rho-associated, coiled-coil containing protein kinase 2
MAPK8IP1	-1.2	0.15	2.5	0.42	Mitogen-activated protein kinase 8 interacting protein 1
RAB29	-1.1	0.01	2.6	0.59	RAB29, member RAS oncogene family
DIAPH1	-1.5	0.01	2.6	0.45	Diaphanous-related formin 1
AP2B1	1.6	0.05	2.6	0.55	Adaptor-related protein complex 2, beta 1 subunit
RAB3C	1.5	0.11	2.6	0.59	RAB3C, member RAS oncogene family
EPS15	0.1	1.62	2.7	0.48	Epidermal growth factor receptor pathway substrate 15
SH3GLB2	-1.2	0.15	2.8	0.58	SH3-domain GRB2-like endophilin B2
ROCK1	1.6	0.02	2.8	0.29	Rho-associated, coiled-coil containing protein kinase 1
VAMP2	-1.3	0.11	2.9	0.53	Vesicle-associated membrane protein 2 (synaptobrevin 2)
