# Supplementary material for: Identification of Host Factors Involved in Human Cytomegalovirus Replication, Assembly, and Egress Using a Two-Step Small Interfering RNA Screen
Source: mBio. 2018 Jun 26;9(3):e00716-18. doi: 10.1128/mBio.00716-18 (PMC6020295; doi:10.1128/mBio.00716-18)
Supplement: TABLE S2 [file mbo003183951st2.pdf]

	PR		VP		
Gene	*Fold change*	*Std dev*	*Fold change*	*Std dev*	*Description*
VAMP2	-1.3	0.11	2.9	0.53	Vesicle-associated membrane protein 2 (synaptobrevin 2)
ROCK1	1.6	0.02	2.8	0.29	Rho-associated, coiled-coil containing protein kinase 1
SH3GLB2	-1.2	0.15	2.8	0.58	SH3-domain GRB2-like endophilin B2
EPS15	0.1	1.62	2.7	0.48	Epidermal growth factor receptor pathway substrate 15
RAB3C	1.5	0.11	2.6	0.59	RAB3C, member RAS oncogene family
AP2B1	1.6	0.05	2.6	0.55	Adaptor-related protein complex 2, beta 1 subunit
DIAPH1	-1.5	0.01	2.6	0.45	Diaphanous-related formin 1
RAB29	-1.1	0.01	2.6	0.59	RAB29, member RAS oncogene family
MAPK8IP1	-1.2	0.15	2.5	0.42	Mitogen-activated protein kinase 8 interacting protein 1
ROCK2	-1.8	0.27	2.5	0.45	Rho-associated, coiled-coil containing protein kinase 2
AMPH	1.1	0.05	2.5	0.50	Amphiphysin
RAB3D	1.3	0.00	2.5	0.39	RAB3D, member RAS oncogene family
GRB2	-1.1	0.03	2.5	0.61	Growth factor receptor-bound protein 2
DNM3	1.1	0.03	2.5	0.63	Dynamin 3
ARF6	-1.4	0.16	2.5	0.36	ADP-ribosylation factor 6
WASF1	-0.3	1.91	2.5	1.12	WAS protein family, member 1
RHOA	1.3	0.06	2.5	0.35	RAS homolog family member A
SYT2	-1.1	0.12	2.5	0.27	Synaptotagmin II
ACTR3	-1.1	0.07	2.4	0.53	ARP3 actin-related protein 3 homolog (yeast)
CYTH3	1.2	0.11	2.4	0.46	Cytohesin 3
TSG101	1.2	0.10	2.4	0.47	Tumor susceptibility 101
ATG12	0.0	1.49	2.4	0.38	Autophagy related 12
ACTR2	-0.1	1.60	2.4	0.28	ARP2 actin-related protein 2 homolog (yeast)
RAB7B	1.3	0.01	2.4	0.29	RAB7B, member RAS oncogene family
RAB11B	-3.2	0.24	2.3	0.41	RAB11B, member RAS oncogene family
ARPC1B	-1.4	0.12	2.3	0.56	Actin related protein 2/3 complex, subunit 1B, 41kDa
CIB3	-1.2	0.09	2.3	0.43	Calcium and integrin binding family member 3
PI4K2A	0.0	1.64	2.3	0.66	Phosphatidylinositol 4-kinase type 2 alpha
PIK3CG	-1.3	0.01	2.3	0.37	Phosphatidylinositol-4,5-bisphosphate 3-kinase, catalytic subunit gamma
EEA1	-1.1	0.06	2.3	0.40	Early endosome antigen 1
VAMP1	-1.7	0.29	2.2	0.48	Vesicle-associated membrane protein 1 (synaptobrevin 1)
VAPA	-1.1	0.12	2.2	0.10	VAMP (vesicle-associated membrane protein)-associated protein A, 33kDa
SNX2	0.1	1.64	2.2	0.07	Sorting nexin 2
RAB3B	-1.2	0.08	2.2	0.16	RAB3B, member RAS oncogene family
ATP6V0A1	-1.8	0.20	2.2	0.50	ATPase, H+ transporting, lysosomal V0 subunit a1
BECN1	-1.2	0.11	2.1	0.41	Beclin 1, autophagy related
CLTCL1	1.5	0.07	2.1	0.22	Clathrin, heavy chain-like 1
PI4KA	-1.3	0.01	2.1	0.27	Phosphatidylinositol 4-kinase, catalytic, alpha
PI4KB	-2.4	0.36	2.1	0.55	Phosphatidylinositol 4-kinase, catalytic, beta
NEDD4	-1.2	0.19	2.1	0.09	Neural precursor cell expressed, developmentally down-regulated 4, E3 ubiquitin protein ligase
CDC42	-2.1	0.04	2.1	0.43	Cell division cycle 42
SEC13	-1.1	0.07	2.0	0.48	SEC13 homolog (S. cerevisiae)
RAB4A	-1.3	0.08	2.0	0.11	RAB4A, member RAS oncogene family
ACACA	-1.3	0.09	2.0	0.26	Acetyl-CoA carboxylase alpha
PICALM	1.5	0.07	2.0	0.29	Phosphatidylinositol binding clathrin assembly protein
WAS	-1.5	0.04	2.0	0.22	Wiskott-Aldrich syndrome
AP1B1	-1.9	0.09	2.0	0.54	Adaptor-related protein complex 1, beta 1 subunit
MAP4K2	-1.4	0.07	2.0	0.55	Mitogen-activated protein kinase kinase kinase kinase 2
AP1M2	1.0	0.02	1.9	0.30	Adaptor-related protein complex 1, mu 2 subunit
STAU1	1.2	0.01	1.9	0.04	Staufen double-stranded RNA binding protein 1
NSF	0.0	1.48	1.9	0.22	N-ethylmaleimide-sensitive factor
VAPB	-1.4	0.05	1.9	0.30	VAMP (vesicle-associated membrane protein)-associated protein B and C
ARPC4	-1.0	0.05	1.9	0.27	Actin related protein 2/3 complex, subunit 4, 20kDa
RAB5B	-1.8	0.20	1.9	0.69	RAB5B, member RAS oncogene family
IP6K3	-1.7	0.18	1.8	0.17	Inositol hexakisphosphate kinase 3
EZR	-1.4	0.27	1.8	0.69	Ezrin
PITPNM1	-1.9	0.12	1.8	0.17	Phosphatidylinositol transfer protein, membrane-associated 1
NEDD4L	1.3	0.01	1.8	0.19	Neural precursor cell expressed, developmentally down-regulated 4-like, E3 ubiquitin protein ligase
TNIK	-1.7	0.26	1.8	0.25	TRAF2 and NCK interacting kinase
CAV1	-1.1	0.01	1.8	0.20	Caveolin 1, caveolae protein, 22kDa
ARPC5	-1.0	0.00	1.7	0.54	Actin related protein 2/3 complex, subunit 5, 16kDa
MAP1LC3A	-1.5	0.12	1.7	0.68	Microtubule-associated protein 1 light chain 3 alpha
LIMK1	1.1	0.05	1.7	0.34	LIM domain kinase 1
RAB5A	-1.1	0.03	1.6	0.25	RAB5A, member RAS oncogene family
PAK1	-2.8	0.55	1.6	0.04	p21 protein (Cdc42/Rac)-activated kinase 1
DNM2	-0.2	1.65	1.6	0.28	Dynamin 2
CAV3	-1.2	0.06	1.6	0.43	Caveolin 3
MAPK8IP3	0.0	1.54	1.5	0.35	Mitogen-activated protein kinase 8 interacting protein 3
AP3D1	0.0	1.46	1.5	0.18	Adaptor-related protein complex 3, delta 1 subunit
SNX1	-1.2	0.12	1.5	0.12	Sorting nexin 1
ARPC2	1.1	0.01	1.5	0.46	Actin related protein 2/3 complex, subunit 2, 34kDa
ITSN2	-3.1	0.27	1.4	0.15	Intersectin 2
HGS	-1.7	0.03	1.4	0.11	Hepatocyte growth factor-regulated tyrosine kinase substrate
AP2A2	-2.0	0.04	1.4	0.14	Adaptor-related protein complex 2, alpha 2 subunit
SYNJ1	-1.4	0.14	1.3	0.13	Synaptojanin 1
GIT1	1.3	0.08	1.3	0.02	G protein-coupled receptor kinase interacting ArfGAP 1
PDCD6IP	-1.5	0.15	1.3	0.21	Programmed cell death 6 interacting protein
SREBF1	-1.3	0.31	1.3	0.06	Sterol regulatory element binding transcription factor 1
CBLC	-1.3	0.03	1.3	0.01	Cbl proto-oncogene C, E3 ubiquitin protein ligase
RAB8A	-1.2	0.06	1.2	0.06	RAB8A, member RAS oncogene family
EPN3	-1.3	0.01	1.2	0.24	Epsin 3
GORASP1	1.1	0.00	1.2	0.00	Golgi reassembly stacking protein 1, 65kDa
AP4E1	-2.3	0.06	1.2	0.21	Adaptor-related protein complex 4, epsilon 1 subunit
CIB1	-2.2	0.20	1.1	0.06	Calcium and integrin binding 1 (calmyrin)
HIP1	-2.0	0.16	1.1	0.01	Huntingtin interacting protein 1
CBL	-1.7	0.32	1.1	0.05	Cbl proto-oncogene, E3 ubiquitin protein ligase
CIB2	-1.3	0.27	1.1	0.08	Calcium and integrin binding family member 2
AP2M1	-1.3	0.09	1.1	0.05	Adaptor-related protein complex 2, mu 1 subunit
ARPC3	-2.4	0.02	0.2	1.75	Actin related protein 2/3 complex, subunit 3, 21kDa
CAV2	-2.0	0.23	0.1	1.80	Caveolin 2
PACSIN1	-1.1	0.03	0.1	1.68	Protein kinase C and casein kinase substrate in neurons 1
CTBP1	-2.4	1.01	0.1	1.79	C-terminal binding protein 1
HIP1R	-1.5	0.31	0.1	1.83	Huntingtin interacting protein 1 related
EPS15L1	-2.4	0.19	0.1	1.88	Epidermal growth factor receptor pathway substrate 15-like 1
PRKD1	-2.3	0.84	0.1	1.70	Protein kinase D1
ARFIP2	1.1	0.03	0.0	1.54	ADP-ribosylation factor interacting protein 2
FASN	-2.5	1.49	0.0	1.47	Fatty acid synthase
CFL1	-1.4	0.42	0.0	1.73	Cofilin 1 (non-muscle)
SYT1	1.1	0.04	0.0	1.67	Synaptotagmin I
VAV2	-1.5	0.12	0.0	1.49	VAV 2 guanine nucleotide exchange factor
CLTA	-1.8	0.17	-0.1	1.66	Clathrin, light chain A
PACSIN3	-2.7	1.18	-0.1	2.65	Protein kinase C and casein kinase substrate in neurons 3
RAB3A	1.1	0.04	-0.1	1.63	RAB3A, member RAS oncogene family
RAB2A	-1.4	0.04	-0.1	1.73	RAB2A, member RAS oncogene family
ARF1	-1.3	0.02	-0.2	1.71	ADP-ribosylation factor 1
DNM1	-1.6	0.04	-0.4	1.97	Dynamin 1
MAPK8IP2	-1.9	0.49	-0.6	2.67	Mitogen-activated protein kinase 8 interacting protein 2
ADAM10	-1.2	0.19	-1.0	3.22	ADAM metallopeptidase domain 10
ARF5	-1.2	0.10	-1.1	0.01	ADP-ribosylation factor 5
CD81	-0.1	1.60	-1.3	0.15	CD81 molecule
EFS	-1.4	0.16	-1.3	0.34	Embryonal Fyn-associated substrate
RAB8B	-1.3	0.17	-1.3	0.07	RAB8B, member RAS oncogene family
CLTC	0.0	1.47	-1.4	0.38	Clathrin, heavy chain (Hc)
RAB11FIP5	1.2	0.04	-1.5	0.18	RAB11 family interacting protein 5 (class I)
RAB1A	-1.7	0.02	-1.5	0.10	RAB1A, member RAS oncogene family
CAMK1	-1.3	0.06	-1.5	0.34	Calcium/calmodulin-dependent protein kinase I
WASF2	-1.7	0.10	-1.5	0.67	WAS protein family, member 2
GNB2L1	-1.8	0.01	-1.6	0.51	Guanine nucleotide binding protein (G protein), beta polypeptide 2-like 1
RAB5C	-1.3	0.05	-1.6	0.08	RAB5C, member RAS oncogene family
EPN1	-1.5	0.16	-1.6	0.68	Epsin 1
RAB6B	-2.4	1.60	-1.7	3.86	RAB6B, member RAS oncogene family
AP2A1	-2.5	0.00	-1.7	0.19	Adaptor-related protein complex 2, alpha 1 subunit
ARF3	-1.9	0.03	-1.7	0.50	ADP-ribosylation factor 3
CLTB	-0.1	1.52	-1.7	0.51	Clathrin, light chain B
PIP5K1A	-1.6	0.03	-1.9	0.54	Phosphatidylinositol-4-phosphate 5-kinase, type I, alpha
EPN2	-1.6	0.57	-2.1	1.26	Epsin 2
SYNJ2	-1.2	0.04	-2.2	0.69	Synaptojanin 2
SNAP91	-3.6	0.13	-2.5	0.08	Synaptosomal-associated protein, 91kDa
SAR1A	-1.1	0.09	-2.5	0.31	Secretion associated, RAS related GTPase 1A
RAB6A	-1.2	0.08	-2.5	1.09	RAB6A, member RAS oncogene family
SH3GLB1	-1.9	0.08	-2.6	0.91	SH3-domain GRB2-like endophilin B1
PI4K2B	-5.3	0.45	-2.8	0.94	Phosphatidylinositol 4-kinase type 2 beta
ARRB2	0.0	1.57	-2.9	1.60	Arrestin, beta 2
RAC1	-1.4	0.24	-3.3	2.01	RAS-related C3 botulinum toxin substrate 1 (Rho family, small GTP binding protein Rac1)
RAB11A	-1.5	0.04	-3.4	1.55	RAB11A, member RAS oncogene family
CBLB	-3.1	0.17	-3.6	0.76	Cbl proto-oncogene B, E3 ubiquitin protein ligase
ATG5	-1.6	0.16	-3.6	0.96	Autophagy related 5
ARRB1	-2.4	0.10	-3.6	0.58	Arrestin, beta 1
AP1M1	-1.2	0.04	-4.2	1.61	Adaptor-related protein complex 1, mu 1 subunit
CLINT1	-1.8	0.13	-4.8	0.60	Clathrin interactor 1
BIN1	-1.1	0.08	-4.9	0.72	Bridging integrator 1
ATM	-1.5	0.20	-5.7	0.82	ATM serine/threonine kinase
WASF3	-2.0	0.55	-6.1	6.81	WAS protein family, member 3
VPS4A	1.1	0.01	-6.4	0.71	Vacuolar protein sorting 4 homolog A (S. cerevisiae)
DAB2	-3.1	0.38	-6.6	2.48	Dab, mitogen-responsive phosphoprotein, homolog 2 (Drosophila)
ITSN1	-1.8	0.27	-6.9	2.09	Intersectin 1 (SH3 domain protein)
RAB4B	1.3	0.37	-7.1	2.29	RAB4B, member RAS oncogene family
FYN	-4.1	1.98	-7.9	4.79	FYN proto-oncogene, Src family tyrosine kinase
ASAP2	-1.6	0.37	-9.3	0.42	ArfGAP with SH3 domain, ankyrin repeat and PH domain 2
PIK3C2G	-2.0	0.83	-9.4	3.34	Phosphatidylinositol-4-phosphate 3-kinase, catalytic subunit type 2 gamma
VPS36	-3.0	0.30	-10.4	0.18	Vacuolar protein sorting 36 homolog (S. cerevisiae)
ERC1	0.0	1.50	-11.3	0.30	ELKS/RAB6-interacting/CAST family member 1
COPA	-1.6	0.07	-12.2	0.44	Coatomer protein complex, subunit alpha
ATP6V0C	-1.5	0.10	-12.3	0.34	ATPase, H+ transporting, lysosomal 16kDa, V0 subunit c
COPB2	-2.0	0.25	-12.3	0.30	Coatomer protein complex, subunit beta 2 (beta prime)
VCP	-4.3	0.70	-12.4	0.49	Valosin containing protein
