# Supplementary material for: Identification of Host Factors Involved in Human Cytomegalovirus Replication, Assembly, and Egress Using a Two-Step Small Interfering RNA Screen
Source: mBio. 2018 Jun 26;9(3):e00716-18. doi: 10.1128/mBio.00716-18 (PMC6020295; doi:10.1128/mBio.00716-18)
Supplement: TABLE S3 [file mbo003183951st3.pdf]

	PR		VP			PR vs VP		
Gene	Fold change	Std dev	Fold change	Std dev	VP / PR ratio	Significance		Description
ERC1	0.0	1.50	-11.3	0.30	0.1	0.009	**	ELKS/RAB6-interacting/CAST family member 1
RAB4B	1.3	0.37	-7.1	2.29	0.1	0.036	*	RAB4B, member RAS oncogene family
ATP6V0C	-1.5	0.10	-12.3	0.34	0.1	0.001	***	ATPase, H+ transporting, lysosomal 16kDa, V0 subunit c
COPA	-1.6	0.07	-12.2	0.44	0.1	0.001	***	Coatomer protein complex, subunit alpha
VPS4A	1.1	0.01	-6.4	0.71	0.1	0.004	**	Vacuolar protein sorting 4 homolog A (S. cerevisiae)
COPB2	-2.0	0.25	-12.3	0.30	0.2	0.001	***	Coatomer protein complex, subunit beta 2 (beta prime)
ASAP2	-1.6	0.37	-9.3	0.42	0.2	0.003	**	ArfGAP with SH3 domain, ankyrin repeat and PH domain 2
PIK3C2G	-2.0	0.83	-9.4	3.34	0.2	0.092	ns	Phosphatidylinositol-4-phosphate 3-kinase, catalytic subunit type 2 gamma
BIN1	-1.1	0.08	-4.9	0.72	0.2	0.018	*	Bridging integrator 1
ATM	-1.5	0.20	-5.7	0.82	0.3	0.020	*	ATM serine/threonine kinase
ITSN1	-1.8	0.27	-6.9	2.09	0.3	0.077	ns	Intersectin 1 (SH3 domain protein)
VPS36	-3.0	0.30	-10.4	0.18	0.3	0.001	**	Vacuolar protein sorting 36 homolog (S. cerevisiae)
AP1M1	-1.2	0.04	-4.2	1.61	0.3	0.122	ns	Adaptor-related protein complex 1, mu 1 subunit
VCP	-4.3	0.70	-12.4	0.49	0.3	0.006	**	Valosin containing protein
CLINT1	-1.8	0.13	-4.8	0.60	0.4	0.021	*	Clathrin interactor 1
ARRB2	0.0	1.57	-2.9	1.60	0.4	0.214	ns	Arrestin, beta 2
SAR1A	-1.1	0.09	-2.5	0.31	0.5	0.027	*	Secretion associated, RAS related GTPase 1A
ATG5	-1.6	0.16	-3.6	0.96	0.5	0.108	ns	Autophagy related 5
RAB11A	-1.5	0.04	-3.4	1.55	0.5	0.228	ns	RAB11A, member RAS oncogene family
DAB2	-3.1	0.38	-6.6	2.48	0.5	0.185	ns	Dab, mitogen-responsive phosphoprotein, homolog 2 (Drosophila)
RAC1	-1.4	0.24	-3.3	2.01	0.5	0.305	ns	RAS-related C3 botulinum toxin substrate 1 (Rho family, small GTP binding protein Rac1)
RAB6A	-1.2	0.08	-2.5	1.09	0.5	0.216	ns	RAB6A, member RAS oncogene family
FYN	-4.1	1.98	-7.9	4.79	0.6	0.403	ns	FYN proto-oncogene, Src family tyrosine kinase
SYNJ2	-1.2	0.04	-2.2	0.69	0.6	0.170	ns	Synaptojanin 2
RAB11FIP5	1.2	0.04	-1.5	0.18	0.6	0.002	**	RAB11 family interacting protein 5 (class I)
CLTB	-0.1	1.52	-1.7	0.51	0.6	0.282	ns	Clathrin, light chain B
ARRB1	-2.4	0.10	-3.6	0.58	0.7	0.096	ns	Arrestin, beta 1
CLTC	0.0	1.47	-1.4	0.38	0.7	0.324	ns	Clathrin, heavy chain (Hc)
SH3GLB1	-1.9	0.08	-2.6	0.91	0.8	0.356	ns	SH3-domain GRB2-like endophilin B1
WASF3	-2.0	0.55	-6.1	6.81	0.8	0.481	ns	WAS protein family, member 3
RAB3A	1.1	0.04	-0.1	1.63	0.8	0.397	ns	RAB3A, member RAS oncogene family
RAB5C	-1.3	0.05	-1.6	0.08	0.8	0.063	ns	RAB5C, member RAS oncogene family
CD81	-0.1	1.60	-1.3	0.15	0.8	0.406	ns	CD81 molecule
SYT1	1.1	0.04	0.0	1.67	0.9	0.425	ns	Synaptotagmin I
CAMK1	-1.3	0.06	-1.5	0.34	0.9	0.437	ns	Calcium/calmodulin-dependent protein kinase I
ADAM10	-1.2	0.19	-1.0	3.22	0.9	0.964	ns	ADAM metallopeptidase domain 10
CBLB	-3.1	0.17	-3.6	0.76	0.9	0.455	ns	Cbl proto-oncogene B, E3 ubiquitin protein ligase
PIP5K1A	-1.6	0.03	-1.9	0.54	0.9	0.519	ns	Phosphatidylinositol-4-phosphate 5-kinase, type I, alpha
EPN2	-1.6	0.57	-2.1	1.26	0.9	0.693	ns	Epsin 2
ARFIP2	1.1	0.03	0.0	1.54	0.9	0.432	ns	ADP-ribosylation factor interacting protein 2
RAB8B	-1.3	0.17	-1.3	0.07	1.0	0.762	ns	RAB8B, member RAS oncogene family
GIT1	1.3	0.08	1.3	0.02	1.0	0.684	ns	G protein-coupled receptor kinase interacting ArfGAP 1
EPN1	-1.5	0.16	-1.6	0.68	1.1	0.923	ns	Epsin 1
EFS	-1.4	0.16	-1.3	0.34	1.1	0.865	ns	Embryonal Fyn-associated substrate
GORASP1	1.1	0.00	1.2	0.00	1.1	0.001	**	Golgi reassembly stacking protein 1, 65kDa
ARF5	-1.2	0.10	-1.1	0.01	1.1	0.237	ns	ADP-ribosylation factor 5
ARF1	-1.3	0.02	-0.2	1.71	1.1	0.452	ns	ADP-ribosylation factor 1
RAB1A	-1.7	0.02	-1.5	0.10	1.1	0.098	ns	RAB1A, member RAS oncogene family
ARF3	-1.9	0.03	-1.7	0.50	1.2	0.650	ns	ADP-ribosylation factor 3
RAB6B	-2.4	1.60	-1.7	3.86	1.2	0.830	ns	RAB6B, member RAS oncogene family
WASF2	-1.7	0.10	-1.5	0.67	1.2	0.771	ns	WAS protein family, member 2
PACSIN1	-1.1	0.03	0.1	1.68	1.2	0.415	ns	Protein kinase C and casein kinase substrate in neurons 1
DNM1	-1.6	0.04	-0.4	1.97	1.2	0.487	ns	Dynamin 1
GNB2L1	-1.8	0.01	-1.6	0.51	1.2	0.516	ns	Guanine nucleotide binding protein (G protein), beta polypeptide 2-like 1
RAB2A	-1.4	0.04	-0.1	1.73	1.3	0.417	ns	RAB2A, member RAS oncogene family
ARPC2	1.1	0.01	1.5	0.46	1.3	0.384	ns	Actin related protein 2/3 complex, subunit 2, 34kDa
CFL1	-1.4	0.42	0.0	1.73	1.3	0.387	ns	Cofilin 1 (non-muscle)
PICALM	1.5	0.07	2.0	0.29	1.4	0.128	ns	Phosphatidylinositol binding clathrin assembly protein
NEDD4L	1.3	0.01	1.8	0.19	1.4	0.066	ns	Neural precursor cell expressed, developmentally down-regulated 4-like, E3 ubiquitin protein ligase
CIB2	-1.3	0.27	1.1	0.08	1.4	0.007	**	Calcium and integrin binding family member 2
AP2M1	-1.3	0.09	1.1	0.05	1.4	0.001	***	Adaptor-related protein complex 2, mu 1 subunit
VAV2	-1.5	0.12	0.0	1.49	1.4	0.307	ns	VAV 2 guanine nucleotide exchange factor
RAB8A	-1.2	0.06	1.2	0.06	1.4	0.001	***	RAB8A, member RAS oncogene family
CLTCL1	1.5	0.07	2.1	0.22	1.4	0.058	ns	Clathrin, heavy chain-like 1
SNAP91	-3.6	0.13	-2.5	0.08	1.4	0.010	**	Synaptosomal-associated protein, 91kDa
MAPK8IP3	0.0	1.54	1.5	0.35	1.5	0.316	ns	Mitogen-activated protein kinase 8 interacting protein 3
AP3D1	0.0	1.46	1.5	0.18	1.5	0.287	ns	Adaptor-related protein complex 3, delta 1 subunit
AP2A1	-2.5	0.00	-1.7	0.19	1.5	0.026	*	Adaptor-related protein complex 2, alpha 1 subunit
SREBF1	-1.3	0.31	1.3	0.06	1.6	0.008	**	Sterol regulatory element binding transcription factor 1
LIMK1	1.1	0.05	1.7	0.34	1.6	0.124	ns	LIM domain kinase 1
EPN3	-1.3	0.01	1.2	0.24	1.6	0.004	**	Epsin 3
MAPK8IP2	-1.9	0.49	-0.6	2.67	1.6	0.557	ns	Mitogen-activated protein kinase 8 interacting protein 2
HIP1R	-1.5	0.31	0.1	1.83	1.6	0.355	ns	Huntingtin interacting protein 1 related
CBLC	-1.3	0.03	1.3	0.01	1.6	0.000	****	Cbl proto-oncogene C, E3 ubiquitin protein ligase
AP2B1	1.6	0.05	2.6	0.55	1.6	0.126	ns	Adaptor-related protein complex 2, beta 1 subunit
CLTA	-1.8	0.17	-0.1	1.66	1.6	0.294	ns	Clathrin, light chain A
STAU1	1.2	0.01	1.9	0.04	1.6	0.001	**	Staufen double-stranded RNA binding protein 1
ROCK1	1.6	0.02	2.8	0.29	1.7	0.027	*	Rho-associated, coiled-coil containing protein kinase 1
RAB5A	-1.1	0.03	1.6	0.25	1.7	0.004	**	RAB5A, member RAS oncogene family
SNX1	-1.2	0.12	1.5	0.12	1.7	0.002	**	Sorting nexin 1
ARPC5	-1.0	0.00	1.7	0.54	1.8	0.019	*	Actin related protein 2/3 complex, subunit 5, 16kDa
RAB3C	1.5	0.11	2.6	0.59	1.8	0.115	ns	RAB3C, member RAS oncogene family
DNM2	-0.2	1.65	1.6	0.28	1.8	0.277	ns	Dynamin 2
AP1M2	1.0	0.02	1.9	0.30	1.9	0.053	ns	Adaptor-related protein complex 1, mu 2 subunit
RHOA	1.3	0.06	2.5	0.35	1.9	0.045	*	RAS homolog family member A
RAB3D	1.3	0.00	2.5	0.39	1.9	0.050	ns	RAB3D, member RAS oncogene family
RAB7B	1.3	0.01	2.4	0.29	1.9	0.034	*	RAB7B, member RAS oncogene family
PDCD6IP	-1.5	0.15	1.3	0.21	1.9	0.004	**	Programmed cell death 6 interacting protein
SYNJ1	-1.4	0.14	1.3	0.13	1.9	0.002	**	Synaptojanin 1
NSF	0.0	1.48	1.9	0.22	1.9	0.217	ns	N-ethylmaleimide-sensitive factor
CAV3	-1.2	0.06	1.6	0.43	1.9	0.012	*	Caveolin 3
CBL	-1.7	0.32	1.1	0.05	1.9	0.006	**	Cbl proto-oncogene, E3 ubiquitin protein ligase
ARPC4	-1.0	0.05	1.9	0.27	2.0	0.004	**	Actin related protein 2/3 complex, subunit 4, 20kDa
TSG101	1.2	0.10	2.4	0.47	2.0	0.073	ns	Tumor susceptibility 101
PI4K2B	-5.3	0.45	-2.8	0.94	2.0	0.080	ns	Phosphatidylinositol 4-kinase type 2 beta
CAV1	-1.1	0.01	1.8	0.20	2.0	0.002	**	Caveolin 1, caveolae protein, 22kDa
SNX2	0.1	1.64	2.2	0.07	2.0	0.207	ns	Sorting nexin 2
CYTH3	1.2	0.11	2.4	0.46	2.1	0.063	ns	Cytohesin 3
FASN	-2.5	1.49	0.0	1.47	2.1	0.229	ns	Fatty acid synthase
PI4K2A	0.0	1.64	2.3	0.66	2.2	0.209	ns	Phosphatidylinositol 4-kinase type 2 alpha
SEC13	-1.1	0.07	2.0	0.48	2.3	0.011	*	SEC13 homolog (S. cerevisiae)
CAV2	-2.0	0.23	0.1	1.80	2.3	0.238	ns	Caveolin 2
HIP1	-2.0	0.16	1.1	0.01	2.3	0.001	**	Huntingtin interacting protein 1
PRKD1	-2.3	0.84	0.1	1.70	2.3	0.222	ns	Protein kinase D1
DNM3	1.1	0.03	2.5	0.63	2.3	0.085	ns	Dynamin 3
HGS	-1.7	0.03	1.4	0.11	2.3	0.001	***	Hepatocyte growth factor-regulated tyrosine kinase substrate
ATG12	0.0	1.49	2.4	0.38	2.3	0.163	ns	Autophagy related 12
AMPH	1.1	0.05	2.5	0.50	2.4	0.054	ns	Amphiphysin
EEA1	-1.1	0.06	2.3	0.40	2.4	0.007	**	Early endosome antigen 1
EPS15	0.1	1.62	2.7	0.48	2.4	0.163	ns	Epidermal growth factor receptor pathway substrate 15
CTBP1	-2.4	1.01	0.1	1.79	2.4	0.229	ns	C-terminal binding protein 1
CIB1	-2.2	0.20	1.1	0.06	2.4	0.002	**	Calcium and integrin binding 1 (calmyrin)
BECN1	-1.2	0.11	2.1	0.41	2.5	0.008	**	Beclin 1, autophagy related
VAPA	-1.1	0.12	2.2	0.10	2.5	0.001	**	VAMP (vesicle-associated membrane protein)-associated protein A, 33kDa
NEDD4	-1.2	0.19	2.1	0.09	2.5	0.002	**	Neural precursor cell expressed, developmentally down-regulated 4, E3 ubiquitin protein ligase
RAB4A	-1.3	0.08	2.0	0.11	2.6	0.001	***	RAB4A, member RAS oncogene family
MAP1LC3A	-1.5	0.12	1.7	0.68	2.6	0.022	*	Microtubule-associated protein 1 light chain 3 alpha
EPS15L1	-2.4	0.19	0.1	1.88	2.6	0.210	ns	Epidermal growth factor receptor pathway substrate 15-like 1
EZR	-1.4	0.27	1.8	0.69	2.6	0.025	*	Ezrin
RAB3B	-1.2	0.08	2.2	0.16	2.6	0.001	**	RAB3B, member RAS oncogene family
ACTR2	-0.1	1.60	2.4	0.28	2.6	0.163	ns	ARP2 actin-related protein 2 homolog (yeast)
ACTR3	-1.1	0.07	2.4	0.53	2.6	0.011	*	ARP3 actin-related protein 3 homolog (yeast)
ACACA	-1.3	0.09	2.0	0.26	2.6	0.003	**	Acetyl-CoA carboxylase alpha
SYT2	-1.1	0.12	2.5	0.27	2.7	0.003	**	Synaptotagmin II
PI4KA	-1.3	0.01	2.1	0.27	2.7	0.003	**	Phosphatidylinositol 4-kinase, catalytic, alpha
AP2A2	-2.0	0.04	1.4	0.14	2.7	0.001	***	Adaptor-related protein complex 2, alpha 2 subunit
AP4E1	-2.3	0.06	1.2	0.21	2.7	0.002	**	Adaptor-related protein complex 4, epsilon 1 subunit
VAPB	-1.4	0.05	1.9	0.30	2.7	0.004	**	VAMP (vesicle-associated membrane protein)-associated protein B and C
CIB3	-1.2	0.09	2.3	0.43	2.7	0.008	**	Calcium and integrin binding family member 3
RAB29	-1.1	0.01	2.6	0.59	2.7	0.013	*	RAB29, member RAS oncogene family
MAP4K2	-1.4	0.07	2.0	0.55	2.7	0.013	*	Mitogen-activated protein kinase kinase kinase kinase 2
PACSIN3	-2.7	1.18	-0.1	2.65	2.8	0.334	ns	Protein kinase C and casein kinase substrate in neurons 3
PIK3CG	-1.3	0.01	2.3	0.37	2.8	0.006	**	Phosphatidylinositol-4,5-bisphosphate 3-kinase, catalytic subunit gamma
GRB2	-1.1	0.03	2.5	0.61	2.9	0.014	*	Growth factor receptor-bound protein 2
ARPC3	-2.4	0.02	0.2	1.75	2.9	0.169	ns	Actin related protein 2/3 complex, subunit 3, 21kDa
WAS	-1.5	0.04	2.0	0.22	2.9	0.002	**	Wiskott-Aldrich syndrome
IP6K3	-1.7	0.18	1.8	0.17	3.0	0.003	**	Inositol hexakisphosphate kinase 3
WASF1	-0.3	1.91	2.5	1.12	3.0	0.215	ns	WAS protein family, member 1
TNIK	-1.7	0.26	1.8	0.25	3.0	0.005	**	TRAF2 and NCK interacting kinase
MAPK8IP1	-1.2	0.15	2.5	0.42	3.1	0.007	**	Mitogen-activated protein kinase 8 interacting protein 1
RAB5B	-1.8	0.20	1.9	0.69	3.2	0.019	*	RAB5B, member RAS oncogene family
SH3GLB2	-1.2	0.15	2.8	0.58	3.3	0.011	*	SH3-domain GRB2-like endophilin B2
ARPC1B	-1.4	0.12	2.3	0.56	3.4	0.011	*	Actin related protein 2/3 complex, subunit 1B, 41kDa
ARF6	-1.4	0.16	2.5	0.36	3.4	0.005	**	ADP-ribosylation factor 6
PITPNM1	-1.9	0.12	1.8	0.17	3.5	0.002	**	Phosphatidylinositol transfer protein, membrane-associated 1
AP1B1	-1.9	0.09	2.0	0.54	3.7	0.010	**	Adaptor-related protein complex 1, beta 1 subunit
VAMP2	-1.3	0.11	2.9	0.53	3.7	0.008	**	Vesicle-associated membrane protein 2 (synaptobrevin 2)
VAMP1	-1.7	0.29	2.2	0.48	3.8	0.010	**	Vesicle-associated membrane protein 1 (synaptobrevin 1)
DIAPH1	-1.5	0.01	2.6	0.45	3.8	0.006	**	Diaphanous-related formin 1
ATP6V0A1	-1.8	0.20	2.2	0.50	3.9	0.009	**	ATPase, H+ transporting, lysosomal V0 subunit a1
CDC42	-2.1	0.04	2.1	0.43	4.2	0.005	**	Cell division cycle 42
ITSN2	-3.1	0.27	1.4	0.15	4.3	0.002	**	Intersectin 2
PAK1	-2.8	0.55	1.6	0.04	4.5	0.008	**	p21 protein (Cdc42/Rac)-activated kinase 1
ROCK2	-1.8	0.27	2.5	0.45	4.6	0.007	**	Rho-associated, coiled-coil containing protein kinase 2
PI4KB	-2.4	0.36	2.1	0.55	4.9	0.011	*	Phosphatidylinositol 4-kinase, catalytic, beta
RAB11B	-3.2	0.24	2.3	0.41	7.5	0.004	**	RAB11B, member RAS oncogene family
